# New Polymorphic Forms of Pemetrexed Diacid and Their Use for the Preparation of Pharmaceutically Pure Amorphous and Hemipentahydrate Forms of Pemetrexed Disodium

**DOI:** 10.3390/molecules200813814

**Published:** 2015-07-30

**Authors:** Olga Michalak, Marta Łaszcz, Kamil Jatczak, Anna Witkowska, Iwona Bujak, Aleksandra Groman, Marcin Cybulski

**Affiliations:** Pharmaceutical Research Institute, 8, Rydygiera Street, 01-793 Warszawa, Poland; E-Mails: m.laszcz@ifarm.eu (M.Ł.); k.jatczak@ifarm.eu (K.J.); a.witkowska@ifarm.eu (A.W.); i.bujak@ifarm.eu (I.B.); a.groman@ifarm.eu (A.G.); m.cybulski@ifarm.eu (M.C.)

**Keywords:** pemetrexed diacid, pemetrexed disodium, powder X-ray diffraction, thermal analysis, phase transition

## Abstract

The preparation of stable amorphous pemetrexed disodium of pharmaceutical purity as well as the process optimization for the preparation of the hemipentahydrate form of pemetrexed disodium are described. Analytical methods for the polymorphic and chemical purity studies of pemetrexed disodium and pemetrexed diacid forms were developed. The physicochemical properties of the amorphous and hydrate forms of pemetrexed disodium, as well as new forms of pemetrexed diacid (a key synthetic intermediate) were studied by thermal analysis and powder X-ray diffraction. High-performance liquid chromatography and gas chromatography methods were used for the chemical purity and residual solvents determination. In order to study the polymorphic and chemical stability of the amorphous and hemipentahydrate forms, a hygroscopicity test (25 °C, 80% RH) was performed. Powder diffraction and high-performance liquid chromatography analyses revealed that the amorphous character and high chemical purity were preserved after the hygroscopicity test. The hemipentahydrate form transformed completely to the heptahydrate form of pemetrexed disodium. Both pemetrexed disodium forms were produced with high efficiency and pharmaceutical purity in a small commercial scale. Amorphous pemetrexed disodium was selected for further pharmaceutical development. Two new polymorphs (forms 1 and 2) of pemetrexed diacid were used for the preparation of high purity amorphous pemetrexed disodium.

## 1. Introduction

An active pharmaceutical ingredient (API) can exist in a solid drug formulation either as a crystalline or amorphous form [1,2]. Among crystalline forms we can distinguish polymorphs and hydrates. The choice of the form which could be useful as an industrial product is determined by many factors resulting from its different properties: melting temperature, stability, solubility, dissolution rate, bioavailability and others, as well as from the manufacturing conditions, the possibility of process control, and also from the restrictions imposed by patents [3,4]. Because each of the particular forms of an API differs in its internal structure, it influences their thermal characteristic or solubility. Each polymorph, hydrate and amorphous form of an individual API also behaves differently under storage conditions. Variations of temperature and humidity substantially affect its stability, giving rise to the following phase transitions: polymorph transformations, hydration/dehydration and crystallization. Regulations concerning these aspects are included in FDA and EMA guidelines [5,6]. Many difficulties can occur during plant scale manufacturing of a desirable form of an API. Even a good protocol for the preparation of the desired form of the active substance and key intermediates, established during an early stage of the drug development, does not preclude difficulties which might be encountered while maintaining desirable forms during the process scale-up. For these reasons, systematic control of an API form has to be performed at every stage of the drug development. Therefore, patents concerning API forms claim their chemical and polymorphic purity as well as manufacturing, synthesis and forms of the key intermediates and impurities. The same concerns drug formulation patents.

**Scheme 1:**
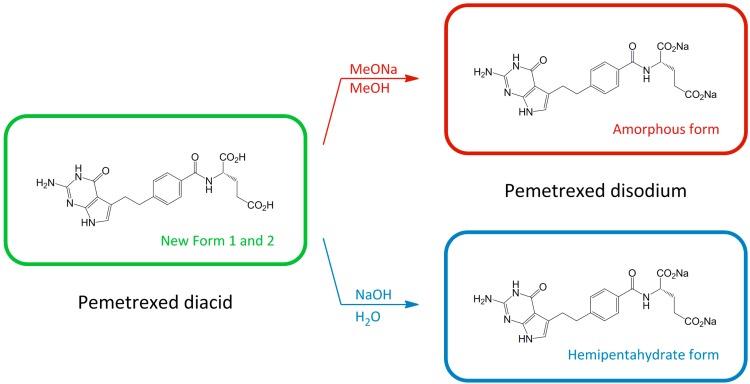
Preparation of amorphous and hemipentahydrate forms of pemetrexed disodium.

Pemetrexed disodium (*N*-[4-[2-(2-amino-4,7-dihydro-4-oxo-1*H*-pyrrolo-[2,3-*d*]-pyrimidin-5-yl)-ethyl]-benzoyl]-L-glutamic acid disodium salt, Scheme 1) belongs to a class of chemotherapeutic drugs known as folate antimetabolites [7,8]. Pemetrexed, developed by Eli Lilly and Company, was approved by the United States Food and Drug administration in 2004 for the treatment of malignant pleural mesothelioma in combination with cisplatin, a platinum-containing chemotherapeutic drug and a second line agent for the treatment of advanced or metastatic non-small cell lung cancer. Currently, the drug is used as a single agent or in combination with other chemotherapeutic agents for the treatment of other types of cancer such as breast cancer, bladder cancer, colorectal carcinoma and cervical cancer [9,10].

Three forms of pemetrexed disodium are currently well established in patent publications. The heptahydrate (HT-PE) form, published in the European Pharmacopoeia [11], is disclosed in the patent publication WO 01/62760 [12]. Both the hemipentahydrate (HP-PE) and amorphous (AM-PE) forms are revealed in several patent publications [12,13,14,15,16,17,18]. A dry powder formulation manufactured through lyophilisation of heptahydrate form has been chosen for the product ALIMTA^®^ [19].

Over the past years, particular attention has been paid to the manufacturing of amorphous pemetrexed disodium salt because of its usefulness in the preparation of a lyophilized pharmaceutical product.

According to the disclosed procedures for the preparation of pemetrexed (AM-PE), the expected amorphous form may be obtained throughout the transformation of pemetrexed diacid or its salts [15,16,17]. The experiments described were generally based on the reaction of specific diacid with a sodium ion source in an aqueous medium followed by removal of water from the solution by distillation under vacuum or atmospheric pressure. However, they never led to morphologically homogeneous pemetrexed disodium. In the patent application [13] it was revealed that the formation of the amorphous pemetrexed disodium phase is accompanied by the formation of a substantial amount of its crystalline phase. Taking into consideration the criteria of pharmaceutical purity and amorphous homogeneity, as well as Good Manufacturing Practice regulatory requirements concerning process validation [20], a simple application of the patent procedure to obtain an amorphous pemetrexed disodium product seemed to be fairly problematic. In order to find more favorable reaction conditions for further pharmaceutical development we undertook a study on the formation of pemetrexed disodium salt (HP-PE and AM-PE).

As a result of our investigations, we described a new processes for the preparation of amorphous pemetrexed disodium (AM-PE) [21,22] as well as an optimized process for the preparation of the HP-PE form. During our attempts to find an efficient and reproducible procedure of the pemetrexed synthesis with an API quality (HP-PE and AM-PE), an analytical study on pemetrexed diacid was performed. As revealed in the patent literature, this commonly known precursor in the synthesis of pemetrexed disodium salt may crystallize in different polymorphic and pseudopolymorphic forms [13,23,24]. In the present work the preparation of two new polymorphs of pemetrexed diacid and a detailed study of their physicochemical properties are discussed.

The quality control of pemetrexed diacid and disodium forms was performed by the following analytical methods: differential scanning calorimetry (DSC), thermogravimetry (TGA), powder X-ray diffraction (PXRD). Water determinations were performed by coulometric Karl Fischer titrations. High-performance liquid chromatography (HPLC) and gas chromatography (GC) methods were used for chemical purity and residual solvents determinations.

## 2. Results and Discussion

### 2.1. Characterization of Pemetrexed Diacid Forms **1** and **2**

#### 2.1.1. Powder X-ray Diffraction

**Figure 1:**
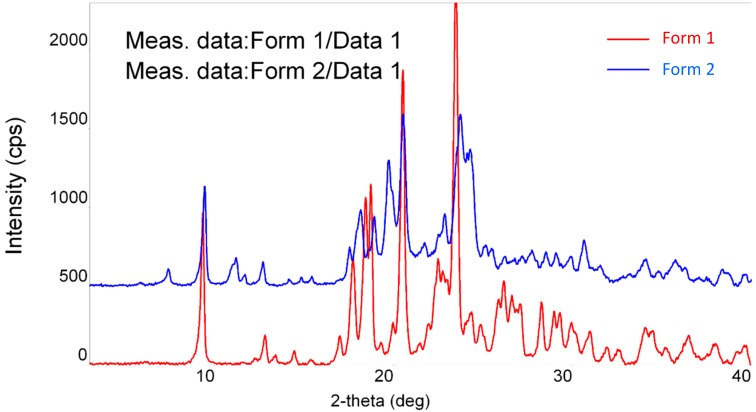
PXRD patterns of pemetrexed diacid forms **1** and **2**.

Diffraction patterns of forms **1** and **2** differ from each other significantly (Figure 1). Characteristic peaks of form **1** are observed at: 9.3; 13.4; 17.0; 17.7; 18.4; 19.3; 28.3 and 31.0° compared to form **2** which has characteristic peaks at: 7.4; 9.4; 11.2; 11.7; 19.7; 23.7 and 27.7°. The PXRD data for both forms differs from that reported in patent literature [13,23,24], (Figures S1–S4 in the Supporting Information).

#### 2.1.2. Thermal Analysis and Degradation Studies

**Figure 2:**
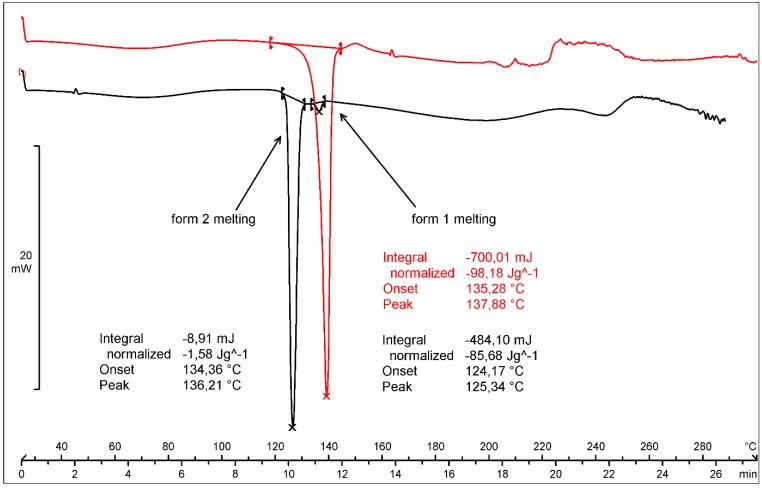
DSC profiles of pemetrexed diacid forms **1** and **2**.

Both DSC curves of forms **1** and **2** are characterized by a broad endothermic effect in the temperature range from 30 to 120 °C connected with the evaporation of residual solvents (Figure 2). Sharp endothermic effects visible at the temperatures of 124.17 and 135.28 °C in the DSC traces come from the melting of form **2** and **1**, respectively. Apart from the main melting effect of form **2**, an additional small (ΔH = −1.58 J/g) effect at 134.34 °C is present in its DSC curve. This is the melting of the seeds of form **1**. Irregular base lines visible in the temperature range from 150 to 300 °C denote the decomposition of the substance. Presumably, in this temperature range DMSO, whose boiling point is 189 °C, evaporates. Additional DSC experiments showed that the transition of form **2** into form **1** is irreversible. Description of these experiments and figures are available in the Supporting Information (Figures S5–S8).

**Figure 3:**
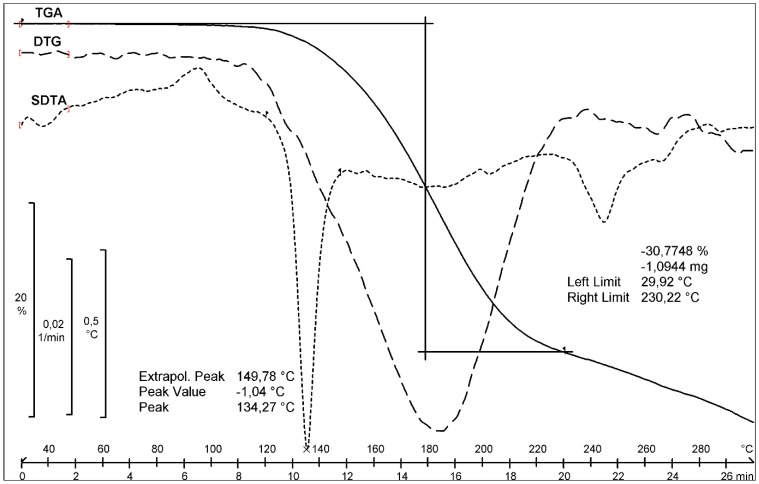
TGA, DTG and SDTA curves of pemetrexed diacid form **1**.

For form **1** the mass loss of 30.77% was evaluated in the temperature range from 30 to 230 °C from the TGA curve (Figure 3).

**Figure 4:**
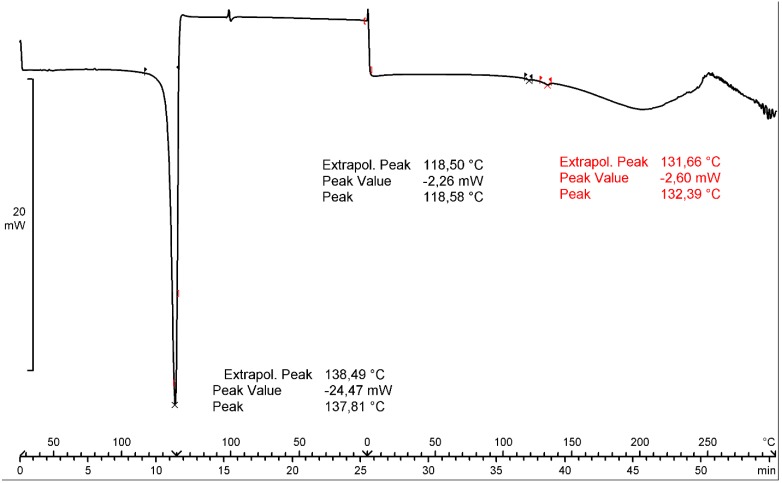
DSC temperature loop of pemetrexed diacid form **1**.

The value of mass loss is coincident with the sum of water (0.49%) and DMSO (30.36%) contents. The comparison of the effects from the TGA, SDTA (provides similar results as the DSC analysis) and DTG curves prove that the endothermic effect visible at 134.27 °C (SDTA curve) is connected with the substance melting, whereas a broad endotherm in the temperature range from 100 to 230 °C (DTG) is connected with the DMSO evaporation. The thermogravimetric behavior of form **2** (Figure S9 in the Supporting Information) is very similar to that of form **1**. It is difficult to unambiguously prove the nature of the DMSO bonding with pemetrexed diacid. An additional DSC experiment was performed in the following temperature loop: heating the sample to 140 °C, then cooling it to 0 °C and heating again to 300 °C (Figure 4). The second heating revealed two small endo-effects in the temperature range from 118 to 132 °C. The melting peak visible in the first heating did not reproduce, which proved the decomposition of the substance with the melting. Such behavior suggests that DMSO is not included in the crystal structure but it is absorbed in form **1**.

Degradation of pemetrexed diacid during heating at temperatures higher than 140 °C was proved by the HPLC analysis. The impurities profile of form **1** changes during heating (at 140 and 150 °C). The sum of impurities increases from 0.62% for the initial sample to 1.24% for the sample heated at 150 °C. Results are collected in Table 1.

To summarize the pemetrexed diacid form studies we can conclude that XRPD and DSC analyses proved the existence of two new polymorphs of pemetrexed diacid. These forms differ from each other in powder diffractograms and melting points (form **1** melts at 135 °C, form **2** at 124 °C). GC analyses revealed that both forms include about 30% of DMSO. This value is in accordance with mass losses values from TGA analyses of both forms. The mass losses for both forms are visible after melting effects what can suggest that DMSO is not bonded in crystal lattices.

**Table 1:** HPLC results of the initial and heated samples of pemetrexed diacid form **1**.

Sample	Impurities	Relative Retention Time	Area, [%]	Sum of Impurities, [%]
	Profile		≥0.05%	
initial sample	Imp. 1	0.08	0.15	0.62
	Imp. 2	1.25	0.12	
heating 1 h/140 °C	Imp. 1	0.08	0.33	1.10
	Imp. 3	0.09	0.06	
	Imp. 4	1.02	0.05	
	Imp. 5	1.21	0.06	
	Imp. 2	1.24	0.13	
heating 1 h/150 °C	Imp. 1	0.08	0.29	1.24
	Imp. 3	0.09	0.05	
	Imp. 5	1.21	0.09	
	Imp. 2	1.23	0.06	
	Imp. 6	1.25	0.15	

### 2.2. Characterization of Pemetrexed Disodium Forms and Their Interconversion Relationship

#### 2.2.1. Powder X-ray Diffraction

**Figure 5:**
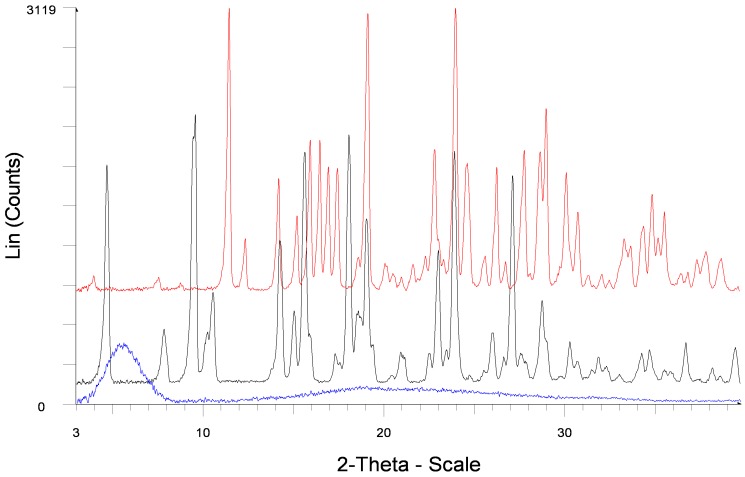
PXRD patterns of HT-PE (**red line**), HP-PE (**black line**) and AM-PE (**blue line**).

The diffraction patterns of HP-PE and HT-PE differ from each other significantly (Figure 5). The characteristic peaks of HP-PE are observed at: 4.7; 9.5; 10.2; 10.5; 18.1 and 27.1° compared to HT-PE which has characteristic peaks at: 4.0; 11.4; 12.3; 16.5; 16.9; 24.6°. The PXRD data for both hydrates matches well with that reported in patent literature [12], (Figure S10 in the Supporting Information).

A broad amorphous halo is observed in the pattern of amorphous pemetrexed. This proves the absence of a long-range inter-molecular order. The PXRD analyses revealed that the amorphous character of the sample AM-PE was preserved after the hygroscopicity test, but HP-PE transformed completely to HT-PE (Figure S11 in the Supporting Information).

Stability studies of HP-PE performed by PXRD after 6 months under following conditions of 25 °C 60% RH and 40 °C 75% RH showed that small peaks from HT-PE are visible on the diffractogram of the sample stored at 25 °C and almost complete transformation to HT-PE in the sample stored at 40 °C (Figure S12 in the Supporting Information). Additional stability studies performed by XRPD for HP-PE under ambient conditions proved that after 1 h of storage a small peak at 11.4° from HT-PE is visible. After 3 h of storage the intensity of this peak increased about three times (Figure S12 in the Supporting Information).

#### 2.2.2. Thermal Analysis

**Figure 6:**
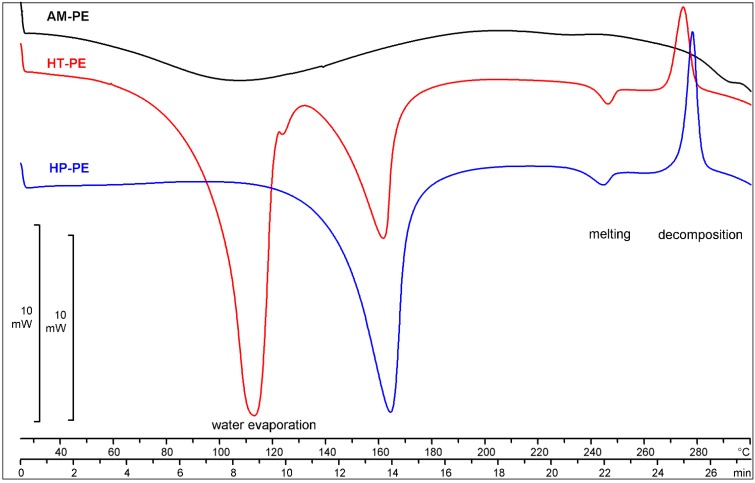
DSC profiles of HP-PE, HT-PE and AM-PE.

In Figure 6 the DSC curves of the hydrates and amorphous pemetrexed are shown. There are three main temperature regions. The first one, from 30 to 220 °C, is connected with water evaporation. The DSC curves differ from each other in this temperature range.

A single broad endothermic effect visible on the DSC curve of AM-PE indicates that water is absorbed in the substance. Narrow endothermic effects visible on the DSC curves of hydrates indicate that water molecules belong to the crystal lattice of the substance. In the second temperature region, about 250 °C, a small endothermic effect coming from the substance melting is visible (no weight change is observed in the TGA curve). In the last temperature region, below 260 °C, exothermic effects show degradation. The TGA curves in this last region are decreasing. The TGA experiments of the hydrates and amorphous pemetrexed were performed from 30 °C to 300 °C (Figures S13 and S14 in the Supporting Information). The mass loss was measured to 220 °C for three forms. The HP-PE was found to lose crystallization water in one step. The mass change of 9.1% is similar to the theoretical stoichiometric ratio of 2.5 mol of water/mol of pemetrexed (8.7%). The HT-PE was found to lose crystallization water in two steps. The sum of mass losses is similar to the theoretical stoichiometric ratio of 7 mol of water/mol of pemetrexed (21.1%). The mass loss connected with the evaporation of the absorbed water and residual solvents from amorphous pemetrexed is 7.8% (Table 2).

The TGA and water content analyses proved that AM-PE and HT-PE are highly hygroscopic after a hygroscopicity test. The water content of the sample AM-PE increased up to 18.5% and the effect of water evaporation visible on the DSC curve of AM-PE became narrower and higher (Figure S15 in the Supporting Information). In spite of high hygroscopic properties, AM-PE preserved its amorphous character and high chemical purity (Table 3).

The TGA analysis of HP-PE after the hygroscopicity test proved that the mass loss increased to 21.3% from the initial value of 9.1%. Two steps of water evaporation are visible on the DSC and TGA curves of HP-PE after the hygroscopicity test, which confirms the transformation into HT-PE (Table 2).

**Table 2:** Results of hygroscopicity tests.

Sample	Increase in Mass according Ph. Eur., [%]	Water Content, [%]	Mass Loss
			TGA, [%]
AM-PE, initial	--	6.5	7.8 *
AM-PE, 24 h 25 °C 80% RH	17.4	18.5	18.5
HP-PE, inital	--	9.0	9.1
HP-PE, 24 h 25 °C 80% RH	21.1	21.3	21.3

* Mass loss is the sum of residual solvents and water content. The content of residual solvents obtained by the GC method is 0.7%.

**Table 3:** HPLC results of the initial studies of AM-PE and HP-PE and those following hygroscopicity tests.

Sample	Impurities	Relative Retention Time	Area, [%]	Sum of Impurities, [%]
	Profile		≥0.05%	
AM-PE, initial	unknown	1.1	0.06	0.34
AM-PE, 24 h 25 °C 80% RH	unknown	1.1	0.05	0.39
HP-PE, initial	--	--	--	0.15
HP-PE, 24 h 25 °C 80% RH	--	--	--	0.22

To summarize the pemetrexed disodium form studies we can conclude that results of XRPD analysis unambiguously showed a lack of a long-range inter-molecular order in amorphous pemetrexed disodium, in contrast to both crystalline hydrate forms. Differences in powder diffractograms of HP-PE and HT-PE are significant because both forms include different number of water molecules in their crystal lattices. It was proved that amorphous character of the sample AM-PE was preserved after higroscopicity studies and HP-PE easily transformed to HT-PE. Stability studies of HP-PE performed by XRPD analysis after 6 months of an accelerated and long term tests also proved its transformation into HT-PE. DSC, TGA techniques are very helpful for the detection of water molecules incorporation into the crystal lattice of HP-PE.

## 3. Experimental Section

### 3.1. Materials

All analytical grade solvents were purchased from Avantor Performance Materials Poland S.A. (Gliwice, Poland) Commercial HT-PE (>99% purity, Pharmasi Chemicals, Shanghai, China) was used as supplied.

#### 3.1.1. Preparation of Pemetrexed Diacid Form **1**

Pemetrexed diethyl ester *p*-toluenesulfonate (922 g, 1.406 mol) was treated with 1.5 M NaOH (aq) (3690 mL) and stirred for 2 h at room temperature. Then ethanol (4170 mL) was added and the mixture was adjusted to pH 3.0 using 1.5 M HCl (aq), then heated to 70–75 °C. The mixture was cooled to room temperature, filtered, and dried in an air flow dryer at 40–45 °C for 24 h to give crude pemetrexed diacid (590 g, HPLC 99.10%) as a blue solid. The crude pemetrexed diacid (590 g) was dissolved in DMSO (1260 mL) at 45–55 °C, then ethanol (4790 mL) was added in one portion and the mixture was stirred for 1 h. The mixture was cooled to room temperature, filtered, and dried under vacuum at 40–45 °C for 72 h to get pemetrexed diacid form 1 (700 g, yield 85%, HPLC 99.48%) as an off-white solid.

#### 3.1.2. Preparation of Pemetrexed Diacid Form **2**

Pemetrexed disodium (278 g) was dissolved in water (1800 mL) and ethanol (2145 mL) was added. The mixture was adjusted to pH 3.0 using 1.5 M HCl (aq), and then heated to 70–75 °C. The mixture was cooled to room temperature, filtered, and dried in an air flow dryer at 40–45 °C for 24 h to get crude pemetrexed diacid (211 g, HPLC 99.19%) as a blue solid. The crude pemetrexed diacid (211 g) was dissolved in DMSO (450 mL) at 45–55 °C, then ethanol (1890 mL) was added in one portion and stirred for 1 h. The mixture was cooled to room temperature, filtered, and dried under vacuum at 40–45 °C for 72 h to get pemetrexed diacid form **2** (230 g, HPLC 99.39%) as an off-white solid. The crude pemetrexed diacid (216 g) was dissolved in DMSO (460 mL) at 45–55 °C. Ethanol was added in one portion (2300 mL) and stirring was continued for about 1 h. The solid was filtered off, washed with ethanol (2 × 500 mL) and dried in an air flow dryer at 40–45 °C (247 g, yield 70%, HPLC 99.52%).

#### 3.1.3. Crystallization of Pemetrexed Diacid Forms **1** and **2**

Pemetrexed diacid can easily be recrystallized by dissolving the solid in an aprotic solvent such as DMSO, DMF or NMP and then precipitating the solid by adding a polar anti-solvent selected form the group of alcohol solvents, preferably EtOH. Pemetrexed diacid can be isolated as any optional crystalline form. We discovered that under these conditions two crystalline forms **1** or **2** of pemetrexed diacid can be obtained and none of them corresponds to the crystalline forms described in the literature [13,23,24]. Crystalline form **1** is obtained when EtOH is added as the anti-solvent to pemetrexed diacid dissolved in DMSO, provided that the EtOH/DMSO volume ratio is maintained from about 2.0 to 4.0. The anti-solvent is added to that solution dropwise or in one portion at the temperature range of 40–55 °C, furnishing the crystalline product precipitation. The formation of crystalline form **1** also takes place when water is added to the mixture of EtOH/DMSO (volume ratio ≤ 2) at the amount not higher than 10% of the total volume. Crystalline form **2** is obtained analogously when EtOH is added as an anti-solvent to pemetrexed diacid dissolved in DMSO at a higher EtOH/DMSO volume ratio, from about 4.2 to 6.0.

#### 3.1.4. Preparation of Amorphous Pemetrexed Disodium

Pemetrexed diacid crystalline forms **1** and **2** of high purity and strictly defined chemical composition are suitable to produce pharmaceutically pure both hemipentahydrate and the amorphous forms of pemetrexed disodium*.* The preparation of the hemipentahydrate form includes crystallization from water and a water soluble solvent. Ultimately, the pure hydrate form was obtained by crystallization from water/ethanol followed by drying under vacuum at 40 °C.

We developed a new process for the preparation of high purity amorphous pemetrexed disodium in the reaction of pemetrexed diacid with sodium cations generating a compound under anhydrous conditions. The amorphous form was obtained by dissolving pemetrexed diacid in the solution of sodium methanolate in methanol at 0–5 °C and followed by adding ethanol or another common organic solvent and drying under moderate temperature.

Pemetrexed diacid form **1** or form **2** (100 g) was added to 0.33 M solution of MeONa in methanol (1000 mL) and stirred at 0–5 °C for 1 h in the atmosphere of inert gas. Then the mixture was filtered and ethanol (1000 mL) was added in one portion. After 0.5 h of stirring at 20–25 °C the mixture was filtered, washed with ethanol (1 × 200 mL), and dried under vacuum for 1.5 h. The crude solid pemetrexed disodium (HPLC 99.61%) was macerated in methanol (250 mL) 3 h at room temperature. The mixture was filtered, washed with cold methanol (1 × 100 mL) and dried under vacuum at 40 °C for 3 h. Next, the solid pemetrexed disodium was macerated in cyclohexane (200 mL) for 3 h at room temperature. The mixture was filtered, washed with cyclohexane (1 × 100 mL) and dried under vacuum at 40 °C for 3 h to get AM-PE (61 g, yield 80%, HPLC 99.71%) as a white solid.

#### 3.1.5. Preparation of Hemipentahydrate Pemetrexed Disodium

Pemetrexed diacid form **1** or form **2** (211 g, HPLC 99.19%) was dissolved in 1 M NaOH (aq) (290 mL) room temperature. The mixture was adjusted to pH 8.0 using 1 M HCl (aq), and then heated to 50–55 °C. Next, ethanol (1200 mL) was added in one portion. The mixture was cooled to room temperature, filtered, and dried under vacuum at 40 °C for 72 h or longer to get HP-PE (143 g, yield 86%, HPLC 99.91%) as an off-white solid.

### 3.2. Methods

#### 3.2.1. Powder X-ray Diffraction

Powder diffractograms were performed on a MiniFlex diffractometer (Rigaku Coporation, Tokyo, Japan) using CuKα1 radiation. The samples were pressed on a glass plate. The instrument was operated in the range from 3 to 40° with the scan rate of 0.02°/min.

#### 3.2.2. Differential Scanning Calorimetry

DSC measurements were performed by means of the DSC822e cell with an IntraCooler (Mettler Toledo GmbH, Schwerzenbach, Switzerland). Samples were weighed as received, without preparation. About 5–10 mg of the studied sample was weighed into a standard aluminium pan (40 µL). The pans were hermetically sealed and perforated before measurements. The samples were heated from 25 to 300 °C at 10 °C/min. The measurements were performed in the nitrogen atmosphere.

#### 3.2.3. Thermogravimetry

TGA measurements were performed by means of the TGA/SDTA851 cell (Mettler Toledo GmbH). About 5–10 mg of the studied sample was weighed into a standard aluminium pan (40 µL). The pans were hermetically sealed and perforated before measurements. The samples were heated from 30 to 300 °C at 10 °C/min in the nitrogen atmosphere. The measurements were blank curve corrected. A TGA analysis provides information on the content of volatile components. DTG is the first derivative of the TGA curve. SDTA is a single differential thermal analysis and provides similar results as the DSC analysis.

#### 3.2.4. Water Determination

The coulometric Karl Fischer titration has been used to determine water content in pemetrexed disodium. Water content was analyzed by the C30 coulometric KF titrator with a Stromboli oven (Mettler Toledo GmbH). The temperature was set as 220 °C. The sample weights were from 0.04 to 0.05 g.

#### 3.2.5. High Performance Liquid Chromatography

A chromatographic analysis was performed using a Waters^®^ Alliance HPLC system (Waters Co. Milford, MA, USA) consisting of the Waters^®^ e2695 Separation Module and Waters^®^ 2998 Photodiode Array Detector. The separation of the analyte from potential impurities was achieved using a Gemini C18 column (150 × 4.6 mm, 3 µm, Phenomenex, Torrance, CA, USA) placed in a thermostated column heater at 25 °C. The mobile phase consisting of A (4 g/L dipotassium hydrogen phosphate; pH 5.2) and B (acetonitrile) was used with a time gradient program T(min)/B (%): 0/5, 8/5, 17/10, 25/10, 31/75, 38/75, 40/5, 46/5 at the flow rate of 0.9 mL/min. Pemetrexed disodium samples were prepared in amber flasks at the concentration of about 0.5 mg/mL (100%) and diluted in the mixture of water/acetonitrile (80/20, *v*/*v*). Pemetrexed diacid samples were prepared at the concentration of about 0.5 mg/mL (100%) and diluted in methanol. The injection volume was 10 µL. The UV detection at 230 nm was used.

#### 3.2.6. Gas Chromatography Methods (a Detailed Description is Provided in the Supporting Information)

Pemetrexed diacid—control of residual dimethyl sulfoxide. GC conditions: gas chromatograph equipped with flame ionization detector and column: DB-WAX (30 m long) with temperature program: 100 °C, ramp 10 °C/min to 220 °C (1 min).

Analysis: standard solution (dimethyl sulfoxide dissolved in dimethylacetamide to obtain a concentration of about 36%) and test solution (*ca.* 10 mg of examined substance dissolved in a 1.0 mL dimethylacetamide) were separately injected into chromatograph. The concentration of dimethyl sulfoxide in% was calculated.

Pemetrexed diacid—control of residual ethanol. GC conditions: gas chromatograph equipped with flame ionization detector, headspace autosampler and column DB-624 (60 m long) with temperature program: 100 °C (6 min), ramp 40 °C/min to 240 °C (5 min).

Analysis: standard solution (1 mL of ethanol dissolved in dimethyl sulfoxide to obtain a concentration of about 0.5% and 1 mL of H_2_O) and test solution (*ca*. 50 mg of examined substance dissolved in a 1 mL of dimethyl sulfoxide and 1 mL of H_2_O) were prepared and headspace injections were performed. The concentration of ethanol in% was calculated.

AM-PE—control of residual methanol, ethanol and cyclohexane. GC conditions: gas chromatograph equipped with flame ionization detector, headspace autosampler and column DB-624 (60 m long), with temperature program: 100 °C (6 min), ramp 40 °C/min to 240 °C (5 min).

Analysis: standard solution I (1 mL of cyclohexane solution: cyclohexane weighed in ethanol and dissolved in dimethyl sulfoxide to obtain a concentration of about 3880 ppm and 1 mL of H_2_O), standard solution II (1 mL of solvents solution (methanol and ethanol dissolved in dimethyl sulfoxide to obtain a concentration of about 3000 ppm and 5000 ppm and 1 mL of H_2_O) and test solution (*ca.* 50 mg of examined substance dissolved in a 1 mL of dimethyl sulfoxide and 1 mL of H_2_O) were prepared and headspace injections were performed. The concentration of solvents in ppm was calculated.

HP-PE—control of residual 4-methylomorpholine, *N*,*N*-dimethylformamide and dimethyl sulfoxide. GC conditions: gas chromatograph equipped with flame ionization detector and column DB-WAX (60 m long) with temperature program: 100 °C, ramp 5 °C/min to 150°C, ramp 10 °C/min to 205 °C, ramp 30 °C/min to 240 °C, 3 min at final temperature.

Analysis: standard solution (analytes dissolved in methanol to obtain a concentration of about 150 ppm of 4-methylomorpholine, 88 mg of *N*,*N*-dimethylformamide and 500 mg of dimethyl sulfoxide) and test solution (*ca.* 30 mg of examined substance dissolved in 1.0 mL of methanol) were separately injected into chromatograph. The areas of peaks of analytes from the test solution must not be bigger than the mean areas from chromatograms obtained with standard solution.

HP-PE—control of residual ethanol, dichloromethane, ethyl acetate and tetrahydrofuran. GC conditions: gas chromatograph equipped with flame ionization detector, headspace autosampler and column DB-624 (60 m long) with temperature program: 120 °C, ramp 2 °C/min to 135 °C, ramp 40 °C/min to 240 °C, 3 min at final temperature.

Analysis: standard solution I (2 mL of ethanol solution: ethanol dissolved in dimethylacetamide to obtain a concentration of about 0.5% and 0.5 mL of H_2_O), standard solution II (2 mL of solvents solution: solvents dissolved in dimethylacetamide to obtain a concentration of about 60 ppm of dichloromethane, 500 ppm of ethyl acetate and 72 ppm of tetrahydrofuran and 0.5 mL of H_2_O) and test solution (*ca.* 50 mg of examined substance was dissolved in a 2 mL of dimethylacetamide and 0.5 mL of H_2_O) were prepared and headspace injections were performed. The concentration of ethanol in% was calculated. The areas of peaks of dichloromethane, ethyl acetate and tetrahydrofuran from the test solution must not be bigger than the mean areas from chromatograms obtained with standard solution II.

#### 3.2.7. Hygroscopicity Test

The studies were performed according to Ph. Eur. chapter 5.11 [25]. HP-PE and AM-PE were stored 24 h under the following conditions of temperature and relative humidity 25 °C ± 1 °C, 80% ± 2% RH. A percentage increase in the mass calculated for HP-PE and AM-PE was 15.6% and 17.4%, respectively.

## 4. Conclusions

The knowledge of polymorphism and relative thermodynamic stability of APIs is very important in the pharmaceutical industry. Polymorphism screening experiments in combination with literature data showed that pemetrexed disodium occurs in three forms: heptahydrate, hemipentahydrate and amorphous ones. The heptahydrate form is used in marketed products. We were able to develop a new process for the preparation of amorphous pemetrexed disodium with the purity requirements for an active substance used in a medicinal product. Amorphous pemetrexed disodium obtained in our study is stable and may be used for the formulations of this important anticancer agent. In this work we also disclosed two new polymorphic forms for pemetrexed diacid which were used for the preparation of high purity amorphous pemetrexed disodium. In this article physicochemical characterizations and the inter-conversion relationship of the crystalline forms and amorphous pemetrexed disodium as well as their intermediate pemetrexed diacid were established using thermoanalytical techniques and powder diffraction.

## Supplementary Materials

Supplementary materials can be accessed at: http://www.mdpi.com/1420-3049/20/08/13814/s1.

## Associated Content

Supporting Information (SI). Detailed description of GC methods. Comparisons of diffractograms from patents with diffractograms of pametrexed diacid and pemetrexed disodium hydrates described in this article. Thermogravimetric curves of pemetrexed diacid of form **2**. Thermogravimetric curves of initial AM-PE and HP-PE samples and after hygroscopicity studies.

## References

[1] Fukuoka E., Makita M., Nakamura Y. (1991). Glassy State of Pharmaceuticals. 5. Relaxation During Cooling and Heating of Glass by Differential Scanning Calorimetry. Chem. Pharm. Bull..

[2] Sidoryk K., Malińska M., Bańkowski K., Kubiszewski M., Łaszcz M., Bodziachowska-Panfil M., Kossykowska M., Giller T., Kutner A., Woźniak K. (2013). Physicochemical characteristics of sunitinib malate and its process-related impurities. J. Pharm. Sci..

[3] Byrn S.R., Pfeiffer R.R., Stowell J.G. (1999). Solid-State Chemistry of Drugs.

[4] Hilfiker R. (2006). Polymorphism in the Pharmaceutical Industry.

[5] (2000). ICH Topic Q 6 A. Specifications: Test Procedures and Acceptance Criteria for New Drug Substances and New Drug Products: Chemical Substances.

[6] FDA (2007). Guidance for Industry, ANDAs: Pharmaceutical Solid Polymorphism. Chemistry, Manufacturing and Controls Information.

[7] Sweetman S.C. (2002). Antineoplastics and immunosuppressants. Martindale—The Complete Drug Reference.

[8] O’Neil M.J. (2006). The Merck Index—An Encyclopedia of Chemicals, Drugs and Biologicals.

[9] Novak K.M. (2005). Drug Facts and Comparisons.

[10] Cohen M.H., Justice R., Pazdur R. (2009). Approval summary: Pemetrexed in the initial treatment of advanced/metastatic non-small cell lung cancer. Oncologist.

[11] (2013). Pemetrexed disodium heptahydrate. European Pharmacopoeia.

[12] Chelius E.C., Reutzel-Edens S.M., Van den Berghe Snorek S. (2001). A Novel Crystalline Form of *N*-[4-[2-(2-amino-4,7-dihydro-4-oxo-3*H*-pyrrolo[2,3-*d*]pyrimidin-5-yl)ethyl]benzoyl]-l-glutamic Acid and Process Therefor. Int. Patent Appl..

[13] Palle R.V., Nariyam S.M., Patel V.B., Vinjamuri R.R.S., Devarakonda S.N., Yarraguntla S.R., Mudapaka V.K., Nalivela V. (2008). Solid Forms of Pemetrexed. Int. Patent Appl..

[14] Li J., Cai Z., Wang M. (2009). Amorphous Polymorph for Pemetrexed Disodium and Preparation Method Thereof. CN Pat. Appl..

[15] Patel N.S., Kilaru S., Thennati R. (2009). Stable Amorphous Form of Pemextred Disodium. Europ. Pat. Appl..

[16] Patel N.S., Kilaru S., Thennati R. (2009). Stable Amorphous Form of Pemetrexed Disodium. US Pat. Appl..

[17] Kadaboina R., Nariyam S.M., Ramakrishnan S., Peddireddy S., Baig M.A., Duggirala N. (2010). Amorphous Pemetrexed Disodium. Int. Patent Appl..

[18] Palle R.V., Nariyam S.M., Patel V.B., Vinjamuri R.R.S., Devarakonda S.N., Yarraguntla S.R., Mudapaka V.K., Nalivela V. (2010). Solid Forms of Pemetrexed. US Pat. Appl..

[19] European Medicines Agency (2004). Protelos: EPAR—Scientific Discussion. http://www.ema.europa.eu/docs/en_GB/document_library/EPAR_Scientific_Discussion/human/000564/WC500025606.pdf.

[20] Good Manufacturing Practice in Pharmaceutical Industry. http://www.pharmacistspharmajournal.org/2011/06/good-manufacturing-practice-in.html.

[21] Michalak O., Jatczak K., Pucko W., Witkowska A., Bujak I., Łaszcz M., Trzcińska K., Kościuch M., Zagrodzka J., Kutner A. (2013). The Preparation Process Development of an Amorphous Form of Pharmaceutically Pure Pemetrexed Disodium. PL Pat. Appl..

[22] Michalak O., Jatczak K., Pucko W., Witkowska A., Łaszcz M., Bujak I., Groman A., Cybulski M. (2014). Process for the Preparation of High Purity Amorphous Pemetrexed Disodium and Crystalline Forms of *N*-[4-[2-(2-Amino-4,7-dihydro-4-oxo-3*H*-pyrrolo[2,3-*d*]pyrimidin-5-yl)ethyl]benzoyl]-l-glutamic Acid. Int. Patent Appl..

[23] Busolli J., Diulgheroff N., Nemethne R.C., Pirkes M., Pontiroli A., Villa M., Aronhime J. (2008). Crystalline Forms of Pemetrexed Diacid and Processes for the Preparation Thereof. Int. Patent Appl..

[24] Luo J., Lin M., Zhu Z., Luo J., Ye W., Qin Y., Deng J. (2010). New Crystalline Forms of Pemetrexed Diacid, and Preparations Thereof. Int. Patent Appl..

[25] (2013). Characters section in monographs. European Pharmacopoeia.

