# Supplementary material for: New Polymorphic Forms of Pemetrexed Diacid and Their Use for the Preparation of Pharmaceutically Pure Amorphous and Hemipentahydrate Forms of Pemetrexed Disodium
Source: Molecules. 2015 Jul 30;20(8):13814–29. doi: 10.3390/molecules200813814 (PMC6332103; doi:10.3390/molecules200813814)
Supplement: Supplementary file 1 [file molecules-20-13814-s001.pdf]

Supporting Information

S1. Description of GC Methods

S1.1. Pemetrexed Diacid

S1.1.1. Control of Residual Dimethyl Sulfoxide by GC Method

GC conditions: gas chromatograph equipped with flame ionization detector and column: DB-WAX (phase composition: polyethylene glycol), film thickeness 0.25 µm, 30 m long, 0.32 mm ID. Temperatures: column 100 °C, ramp 10 °C/min to 220 °C (1 min); injecton port 240 °C; detector 260 °C. Carrier gas: nitrogen, pressure 100 kPa, split 20:1.

Analysis: 1 µL of *standard solution* (dimethyl sulfoxide dissolved in dimethylacetamide to obtain a concentration of about 35% with respect to sample preparation) and 1 µL of *test solution* (*ca.* 10 mg of examined substance dissolved in a 1.0 mL dimethylacetamide) were separately injected into chromatograph. The concentration of dimethyl sulfoxide in % was calculated.

S1.1.2. Control of Residual Ethanol by GC-HS Method

GC conditions: gas chromatograph equipped with flame ionization detector and column: DB-624 (phase composition: 6% cyanopropylphenyl—94% dimethylpolysiloxane), film thickeness 1.8 µm, 60 m long, 0.32 mm ID. Temperatures: column 100 °C (6 min), ramp 40 °C/min to 240 °C (5 min); injecton port 240 °C; detector 260 °C. Carrier gas: nitrogen, pressure 100 kPa, split 5:1. *Headspace autosampler conditions*: temperatures: vial oven 95 °C, needle 110 °C, transfer line 120 °C; thermostat: 30 min, inject: 0.05 min.

Analysis: separate headspace vials containing *standard solution* and *test solution* were prepared and headspace injection was performed. The concentration of ethanol in % was calculated.

Standard solution: 1 mL of ethanol solution (ethanol dissolved in dimethyl sulfoxide to obtain a concentration of about 0.5% with respect to sample preparation) and 1 mL of H_2O.

Test solution: *ca.* 50 mg of examined substance dissolved in a 1 mL of dimethyl sulfoxide and 1 mL of H_2O.

S2. AM-PE

S2.1. Control of Residual Methanol, Ethanol and Cyclohexane by GC-HS Method

GC conditions: gas chromatograph equipped with flame ionization detector and column: DB-624 (phase composition: 6% cyanopropylphenyl—94% dimethylpolysiloxane), film thickeness 1.8 µm, 60 m long, 0.32 mm ID. Temperatures: column 100 °C (6 min), ramp 40 °C/min to 240 °C (5 min); injecton port 240 °C; detector 260 °C. Carrier gas: nitrogen, pressure 100 kPa, split 5:1. *Headspace autosampler conditions*: temperatures: vial oven 95 °C, needle 110 °C, transfer line 120 °C; thermostat: 30 min, inject: 0.05 min.

Analysis: separate headspace vials containing *standard solution I, standard solution II* and *test solution* were prepared and headspace injections were performed. The concentration of solvents in ppm was calculated.

Standard solution I: 1 mL of cyclohexane solution (cyclohexane weighed in ethanol and dissolved in dimethyl sulfoxide to obtain a concentration of about 3880 ppm with respect to sample preparation) and 1 mL of H_2O.

Standard solution II: 1 mL of solvents solution (methanol and ethanol dissolved in dimethyl sulfoxide to obtain a concentration of about 3000 ppm of methanol and 5000 ppm of ethanol with respect to sample preparation) and 1 mL of H_2O.

Test solution: *ca.* 50 mg of examined substance dissolved in a 1 mL of dimethyl sulfoxide and 1 mL of H_2O.

S3. HP-PE

S3.1. Control of Residual 4-Methylomorpholine, N,N-Dimethylformamide and Dimethyl Sulfoxide by GC Method

GC conditions: gas chromatograph equipped with flame ionization detector and column: DB-WAX (phase composition: polyethylene glycol), film thickeness 0.50 µm, 60 m long, 0.32 mm ID. Temperatures: column 100 °C, ramp 5 °C/min to 150 °C, ramp 10 °C/min to 205 °C, ramp 30 °C/min to 240 °C, 3 min at final temperature; injecton port 220 °C; detector 260 °C. Carrier gas: nitrogen, pressure 100 kPa, split 5:1.

Analysis: 1 µL of *standard solution* (analytes dissolved in methanol to obtain a concentration of about 150 ppm of 4-methylomorpholine, 88 mg of *N*,*N*-dimethylformamide and 500 mg of dimethyl sulfoxide with respect to sample preparation) and 1 µL of *test solution* (*ca.* 30 mg of examined substance dissolved in 1.0 mL of methanol) were separately injected into chromatograph. The areas of peaks of analytes from the test solution must not be bigger than the mean areas from chromatograms obtained with standard solution.

S3.2. Control of Residual Ethanol, Dichloromethane, Ethyl Acetate and Tetrahydrofuran by GC-HS Method

GC conditions: gas chromatograph equipped with flame ionization detector and column: DB-624 (phase composition: 6% cyanopropylphenyl—94% dimethylpolysiloxane), film thickeness 1.8 µm, 60 m long, 0.32 mm ID. Temperatures: column 120 °C, ramp 2 °C/min to 135 °C, ramp 40 °C/min to 240 °C, 3 min at final temperature; injecton port 240 °C; detector 260 °C. Carrier gas: nitrogen, pressure 100 kPa, split 5:1. *Headspace autosampler conditions*: temperatures: vial oven 95 °C, needle 110 °C, transfer line 120 °C; thermostat: 30 min, inject: 0.05 min.

Analysis: separate headspace vials containing *test solution*, *standard solution I* and *standard solution II* were prepared and headspace injections were performed. The concentration of ethanol in % was calculated. The areas of peaks of dichloromethane, ethyl acetate and tetrahydrofuran from the test solution must not be bigger than the mean areas from chromatograms obtained with standard solution II.

Standard solution I: 2 mL of ethanol solution (ethanol dissolved in dimethylacetamide to obtain a concentration of about 0.5% with respect to sample preparation) and 0.5 mL of H_2O.

Standard solution II: 2 mL of solvents solution (solvents dissolved in dimethylacetamide to obtain a concentration of about 60 ppm of dichloromethane, 500 ppm of ethyl acetate and 72 ppm of tetrahydrofuran with respect to sample preparation) and 0.5 mL of H_2O.

Test solution: *ca.* 50 mg of examined substance was dissolved in a 2 mL of dimethylacetamide and 0.5 mL of H_2O.

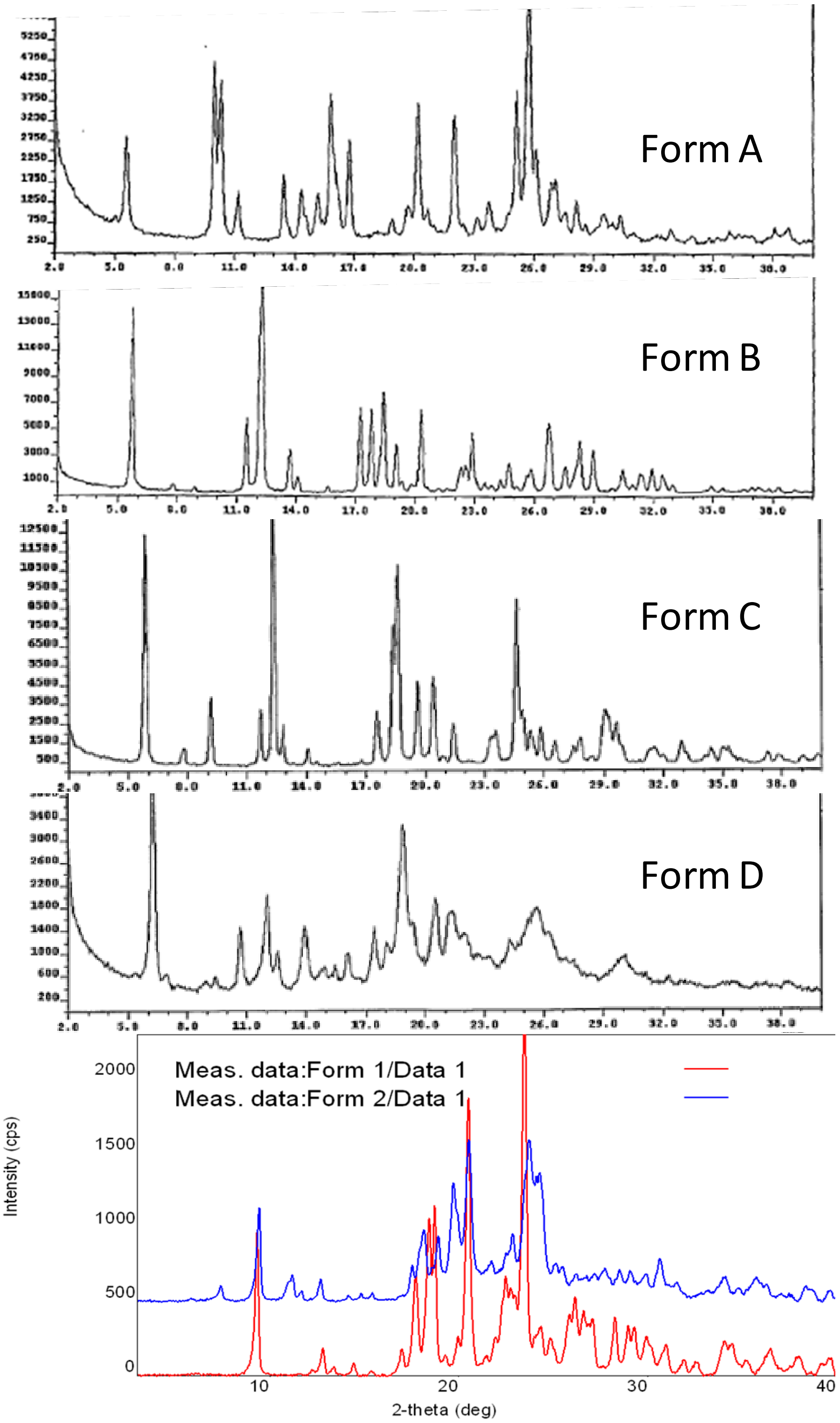

Figure S1. Pemetrexed diacid difractograms comparison of forms: **A**, **B**, **C** and **D** from Int. Patent Appl. WO 2008/021405 A1 with diffractograms of forms 1 and 2.

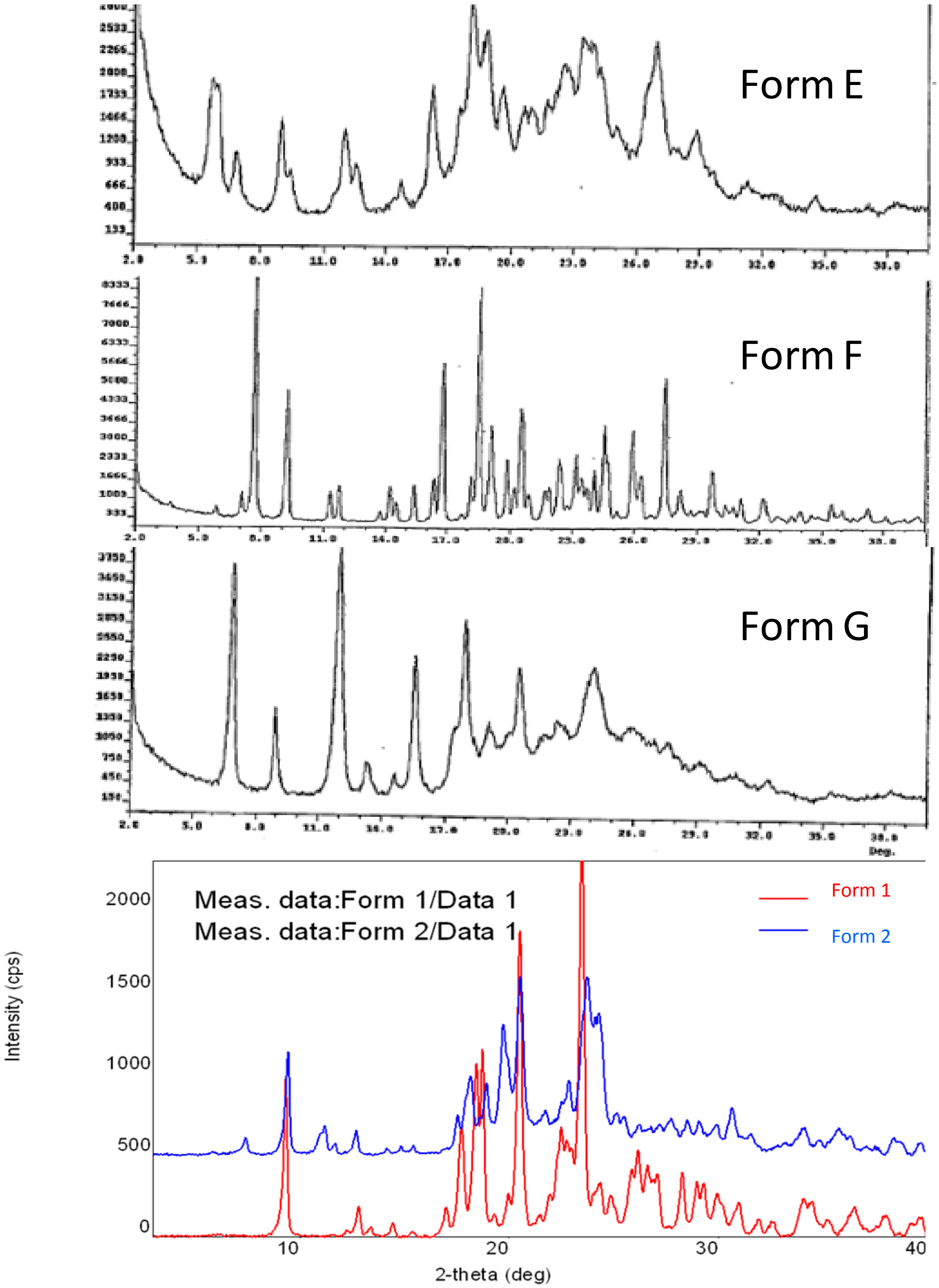

Figure S2. Pemetrexed diacid difractograms comparison of forms: **E**, **F** and **G** from Int. Patent.

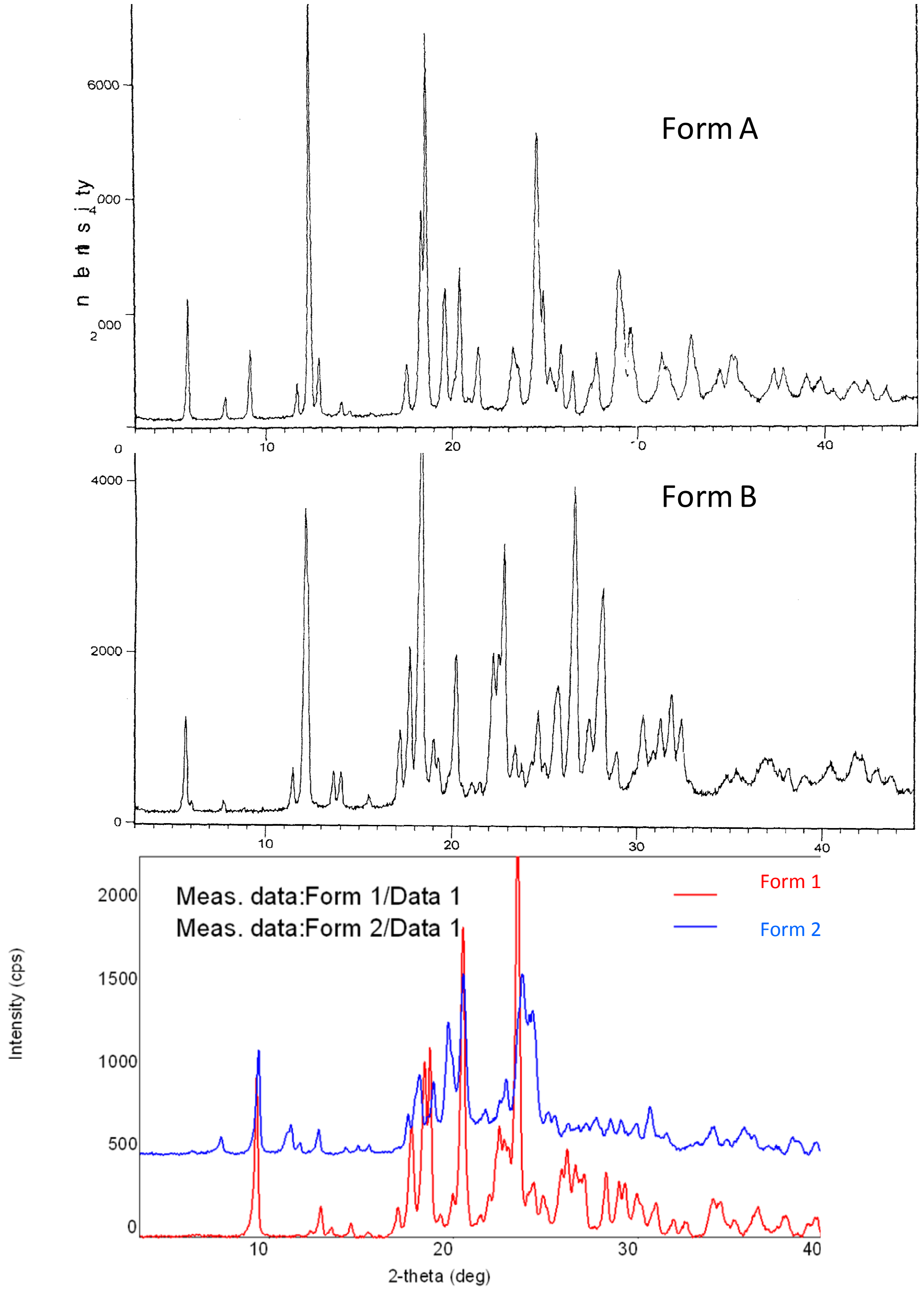

Figure S3. Pemetrexed diacid difractograms comparison of forms **A** and **B** from Int. Patent Appl. WO 2008/124485 A2 with diffractograms of forms 1 and 2.

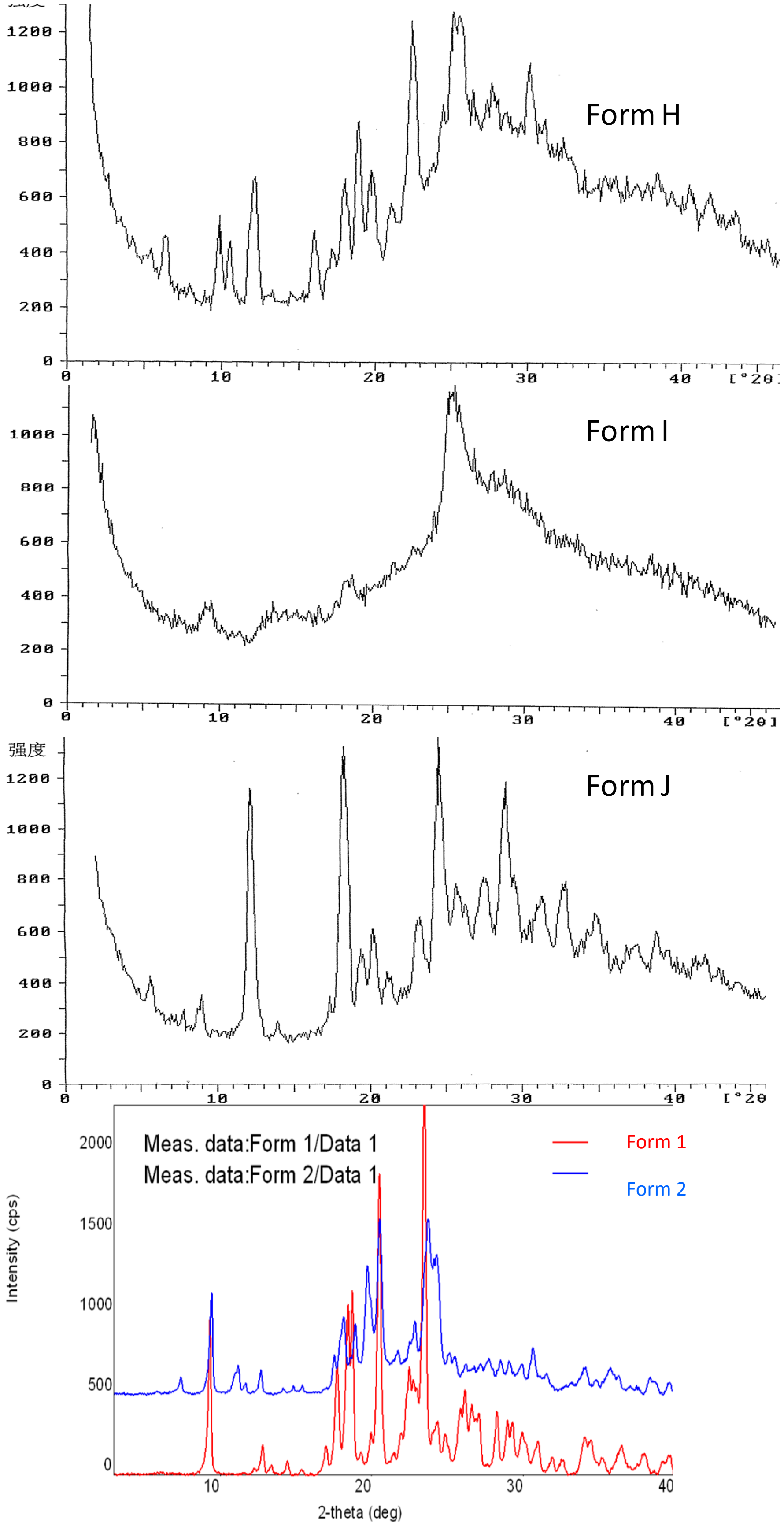

Figure S4. Pemetrexed diacid difractograms comparison of forms: **H**, **I** and **J** from Int. Patent Appl. WO 2010/031357 A1 with diffractograms of forms 1 and 2.

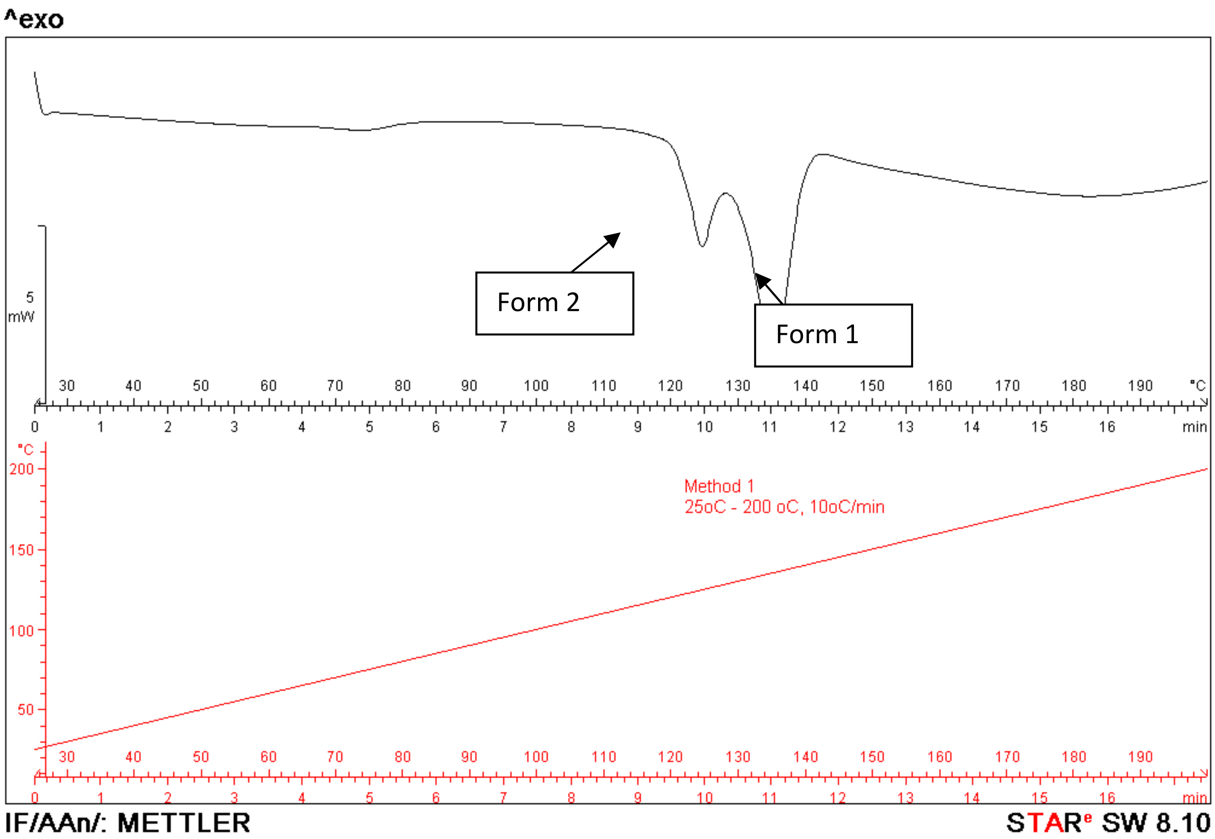

Figure S5. DSC curve of the mixture of forms **1** and **2** and temperature program for Method 1.

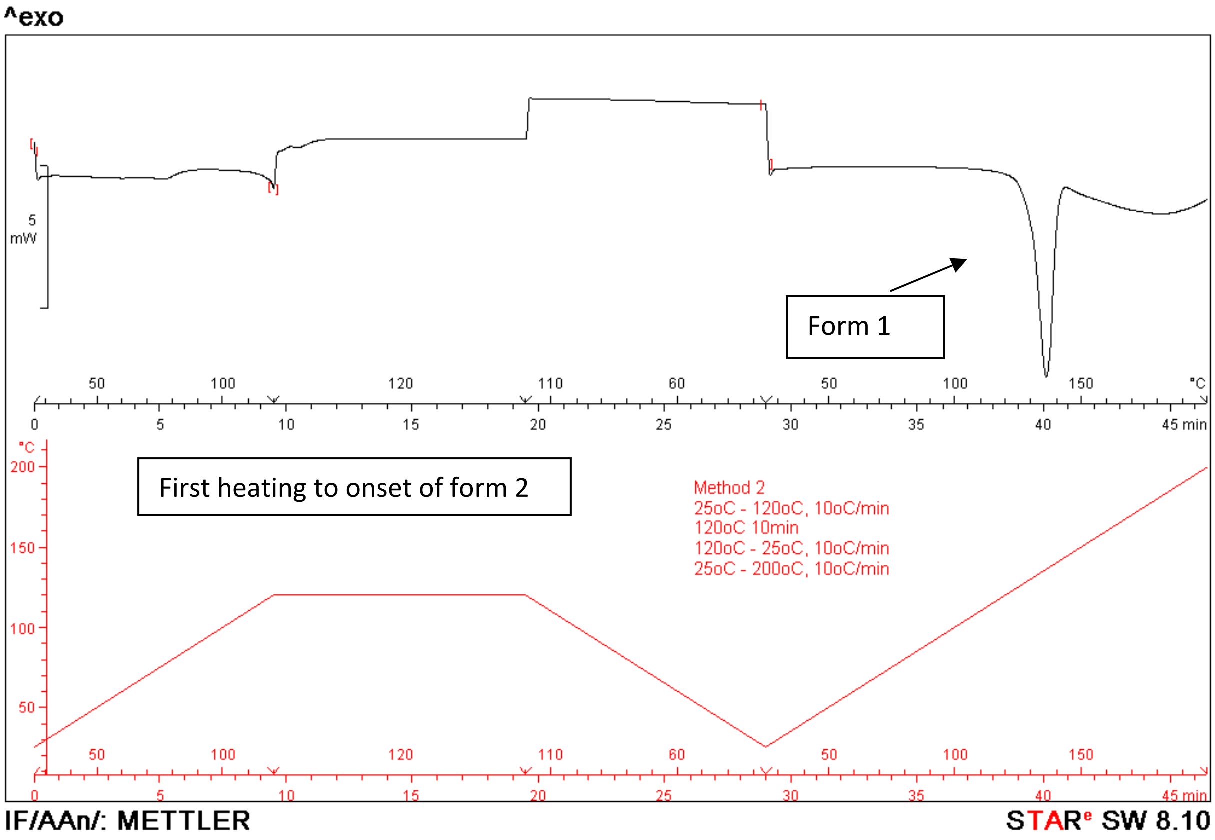

Figure S6. DSC curve of the mixture of forms **1** and **2** and temperature program for Method 2.

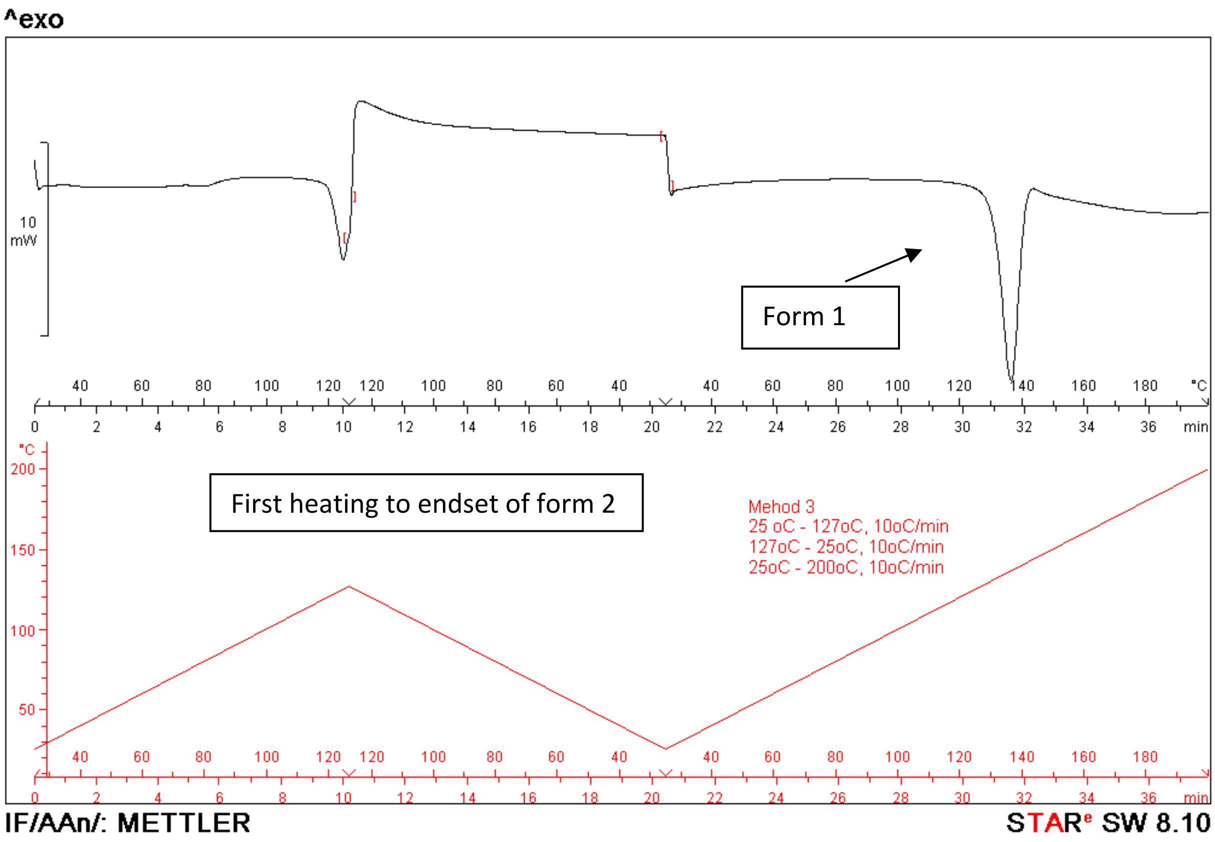

Figure S7. DSC curve of the mixture of forms **1** and **2** and temperature program for Method 3.

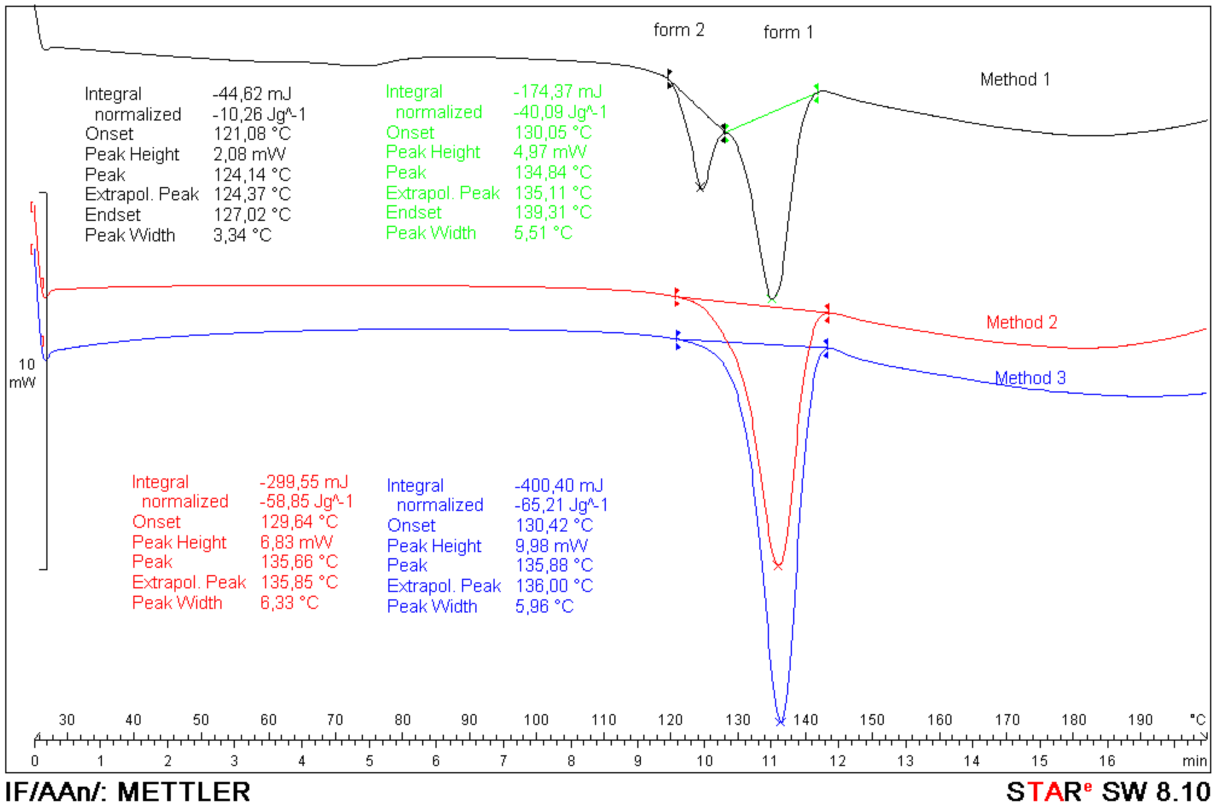

Figure S8. The collection of DSC curves of the mixture of forms **1** and **2** from the heating range of 25–200 °C.

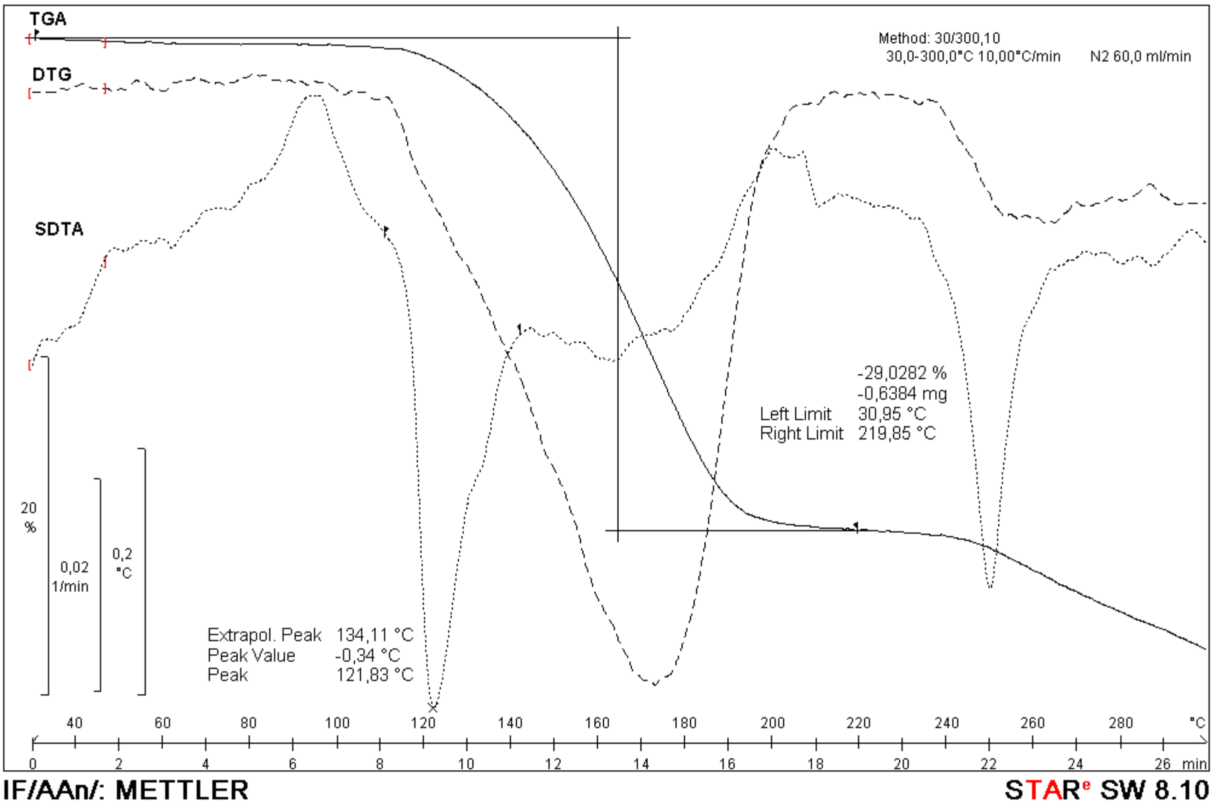

Figure S9. TGA, DTG and SDTA curves of pemetrexed diacid of form 2.

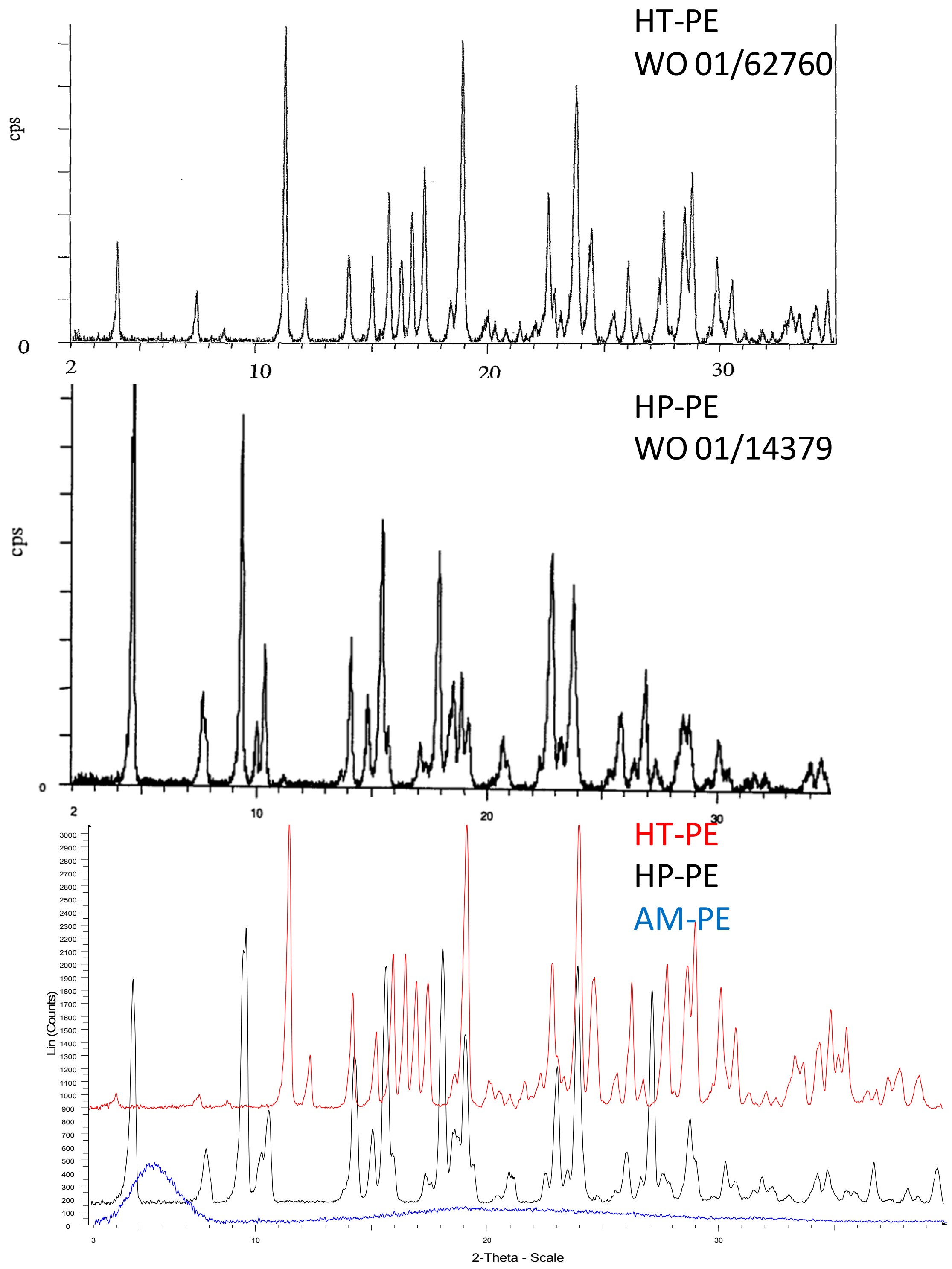

Figure S10. Difractograms comparison of pemetrexed disodium heptahydrate (HT-PE), hemipentahydrate (HP-PE) and amorphous form (AM-PE).

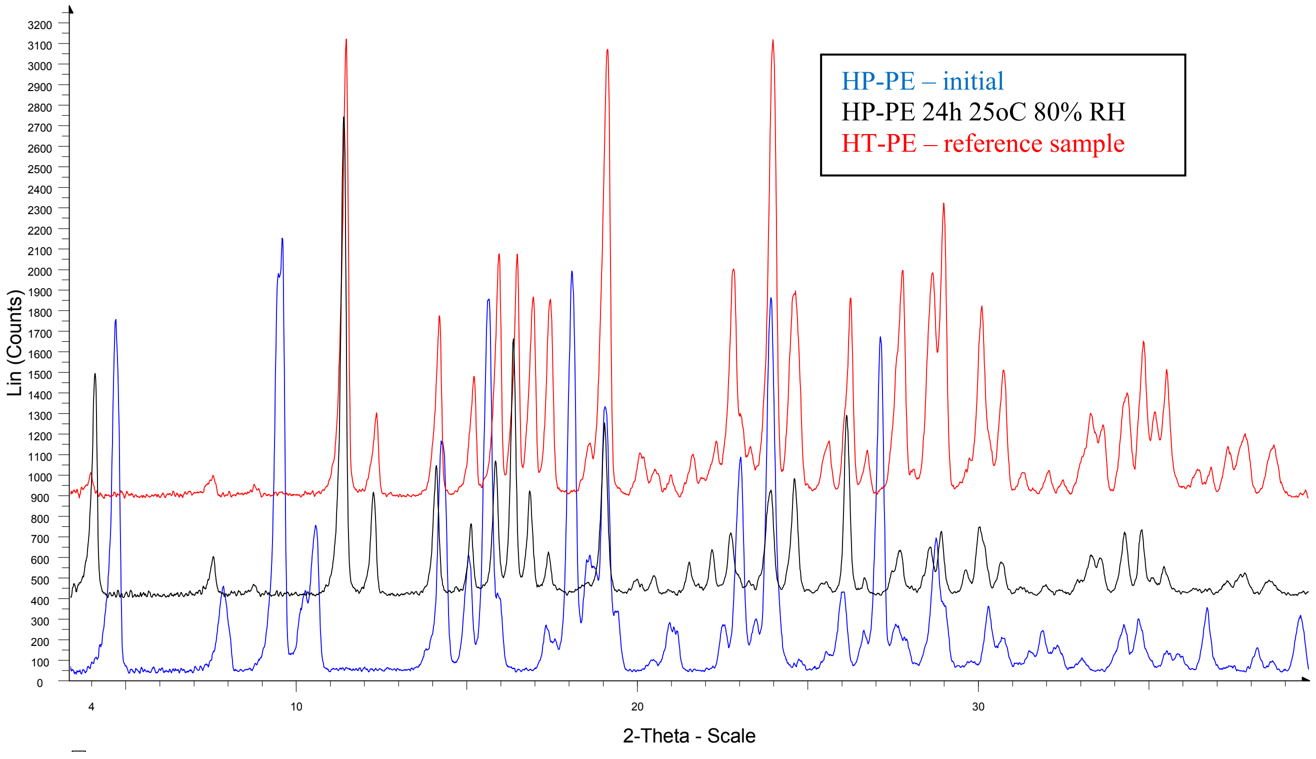

Figure S11. Difractograms comparison of pemetrexed disodium heptahydrate (HT-PE) before and after hygroscopicity test.

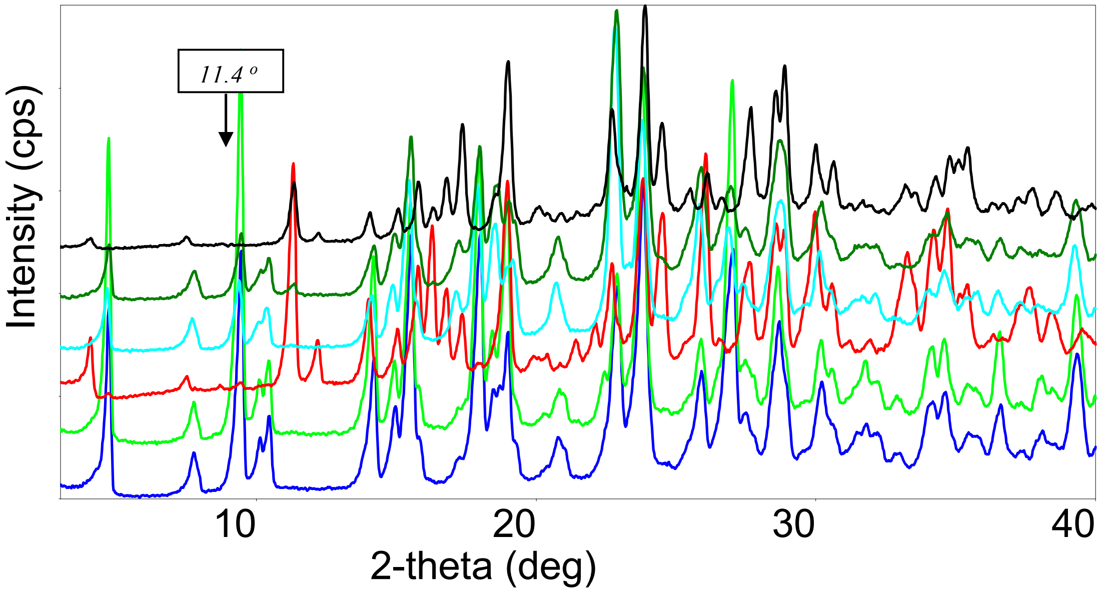

**Samples were packed in two polyethylene bags placed in aluminium/PCV laminated foil and tightly closed by welding. Such packed material was placed inside a cylindrical, cardboard boxes.*

Figure S12. The diffractograms comparison of HP-PE—initial (–), HP-PE stored 6 months under 25 °C and 60% RH* (–), HP-PE stored 6 months under 40 °C and 75 % RH* (–), HP-PE stored 1 h under ambient conditions (–), HP-PE stored 3 h under ambient conditions (–), HT-PE—reference standard (–). The characteristic peak from HT-PE visible in powder patterns of stored samples is indicated by arrow.

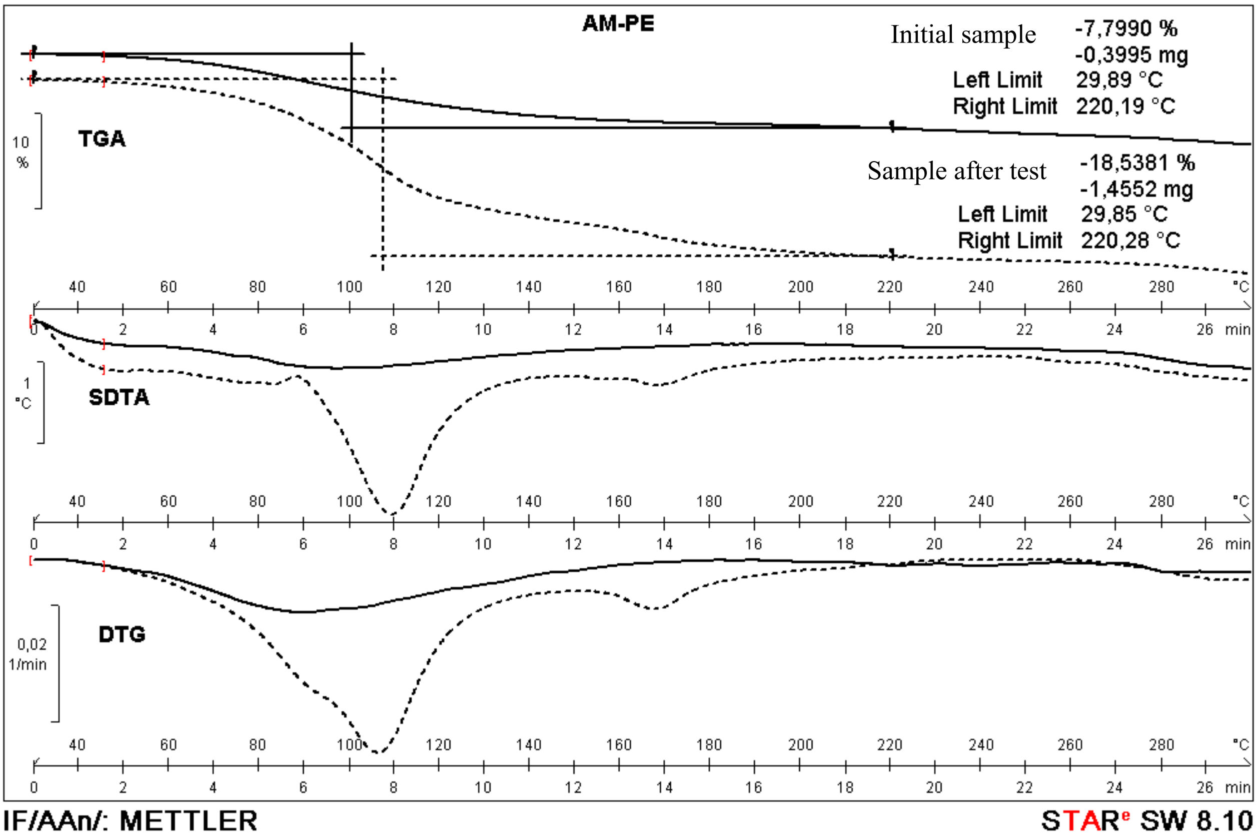

Figure S13. TGA, SDTA and DTG curves of initial AM-PE (plain line) and after higroscopicity test (dotted line).

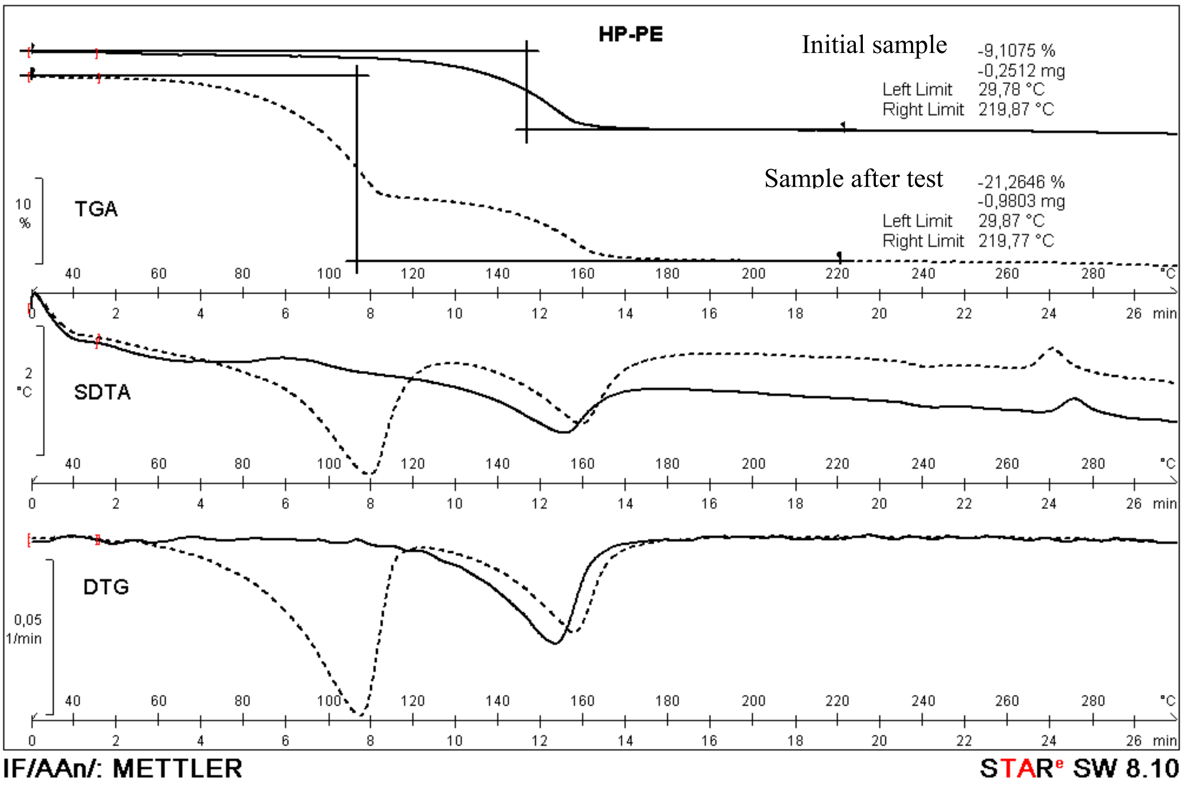

Figure S14. TGA, SDTA and DTG curves of initial HP-PE (plain line) and after higroscopicity test (**dotted line**).

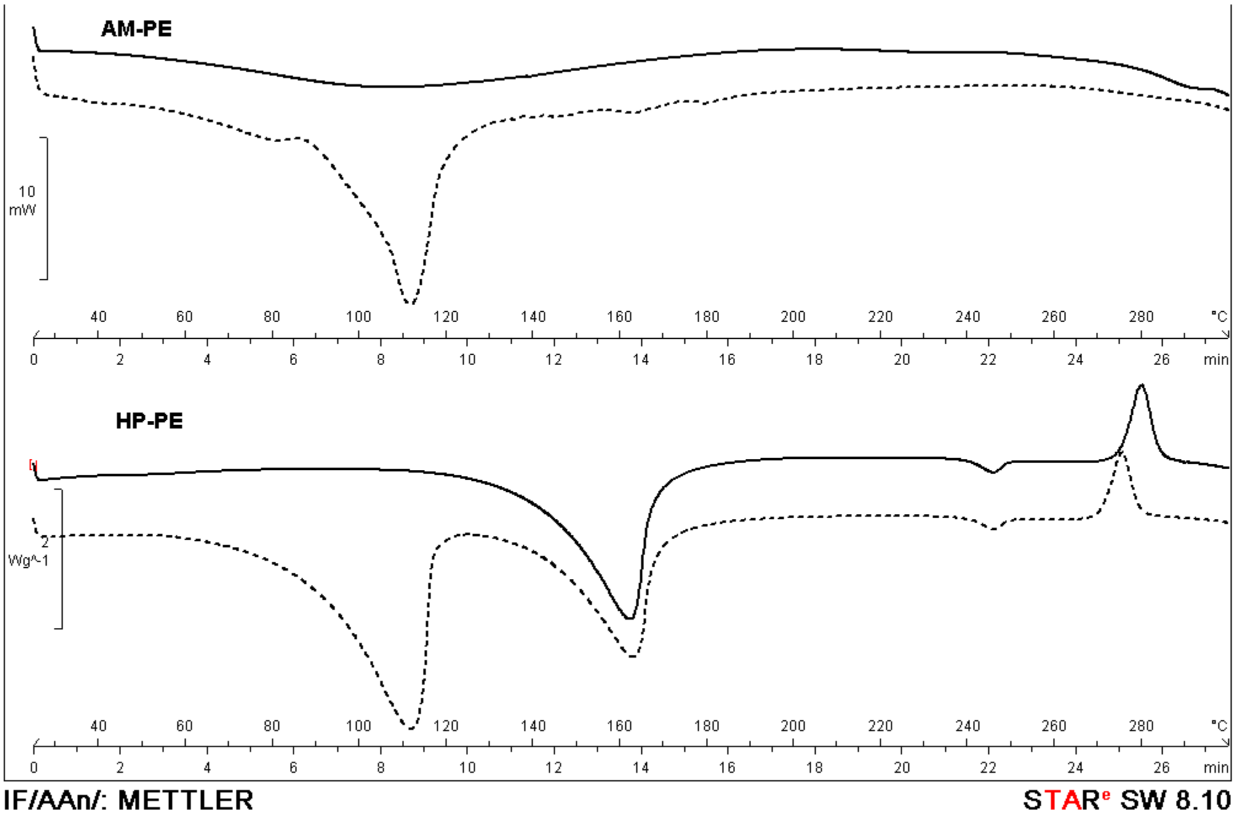

Figure S15. DSC curves of initial AM-PE and HP-PE and after higroscopicity test (**dotted line**).

Results of elemental analysis performed on pemetrexed disodium hemipantahydrate (HP-PE) and heptahydrate (HT-PE), confirm assumed element percentage compositions in the samples.

(HP-PE) $C_{20}H_{19}N_5Na_2O_6 \cdot 2.5H_2O$, molecular weight 516.39.

Table S1. Elemental analysis results of HP-PE.

Element	Theoretical Compositions, %	Mean Result of Elementary Analysis, %
C	46.52	46.57
H	4.68	4.61
N	13.56	13.52

(HT-PE) $C_{20}H_{19}N_5Na_2O_6 \cdot 7H_2O$, molecular weight 597.49.

Table S2. Elemental analysis results of HT-PE.

Element	Theoretical Compositions, %	Mean Result of Elementary Analysis, %
C	40.20	40.29
H	5.57	5.49
N	11.72	11.55

DMSO bands are very well visible in FTIR spectra of both forms.

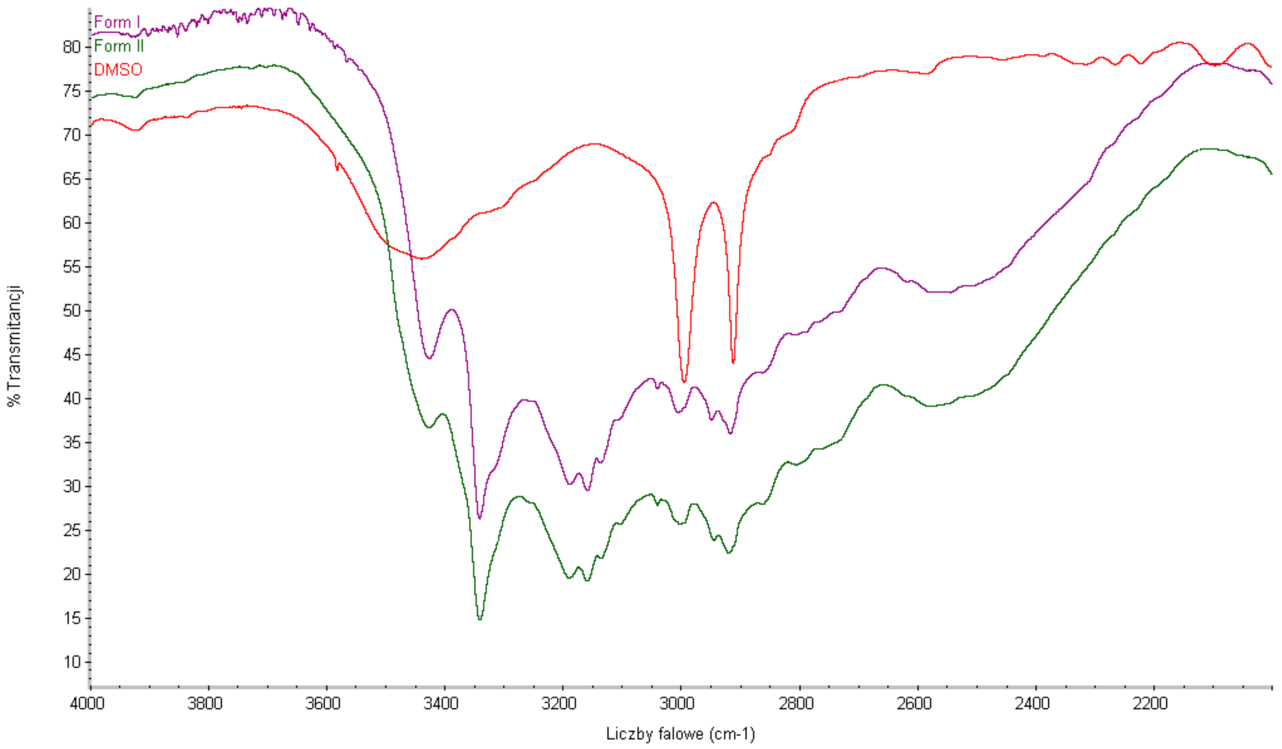

Figure S16a. The FTIR spectra comparinon of forms **I** and **II** with DMSO.

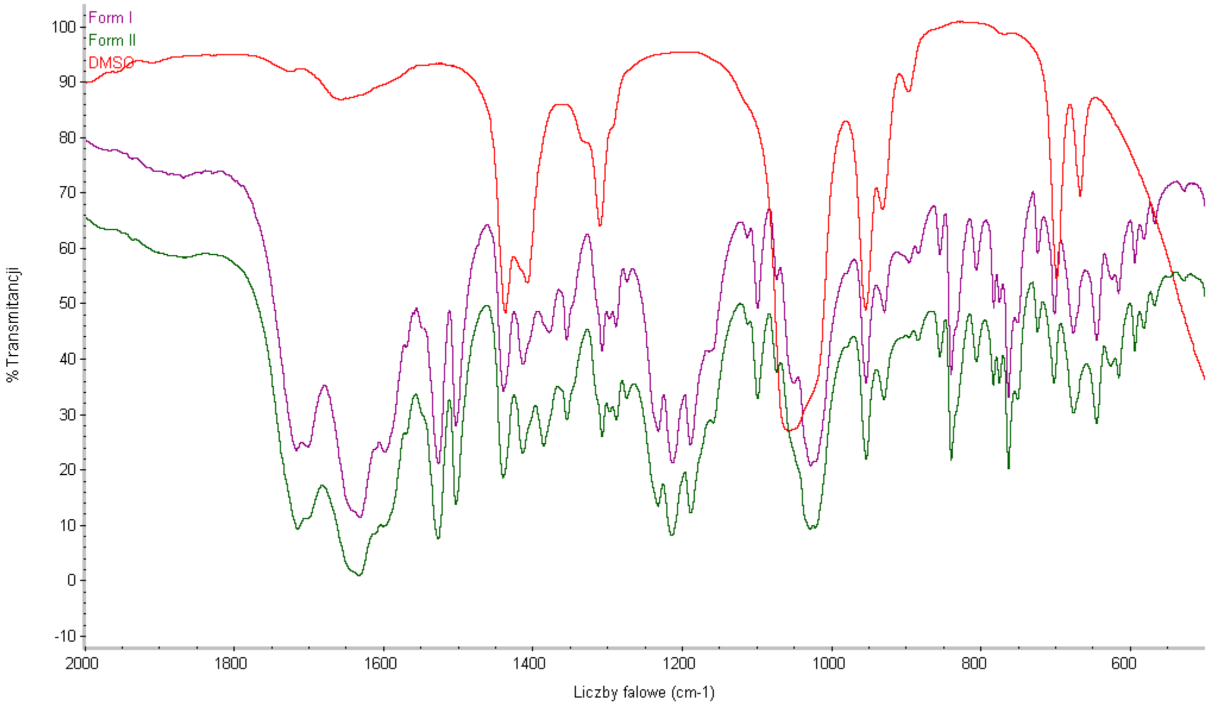

Figure S16b. The FTIR spectra comparinon of forms **I** and **II** with DMSO.
